# Enhanced procedures for mosquito identification by MALDI-TOF MS

**DOI:** 10.1186/s13071-022-05361-0

**Published:** 2022-06-30

**Authors:** Roland Bamou, Monique Melo Costa, Adama Zan Diarra, Ademir Jesus Martins, Philippe Parola, Lionel Almeras

**Affiliations:** 1 Aix-Marseille Univ., IRD, SSA, AP-HM, VITROME, Marseille, France; 2 IHU Méditerranée Infection, Marseille, France; 3 Unité Parasitologie et Entomologie, Département Microbiologie et Maladies Infectieuses, Institut de Recherche Biomédicale des Armées, Marseille, France; 4 Laboratório de Fisiologia e Controle de Artrópodes Vetores, Instituto Oswaldo Cruz (FIOCRUZ), Rio de Janeiro, RJ Brazil; 5 Instituto Nacional de Ciência e Tecnologia em Entomologia Molecular (INCT-EM), Universidade Federal do Rio de Janeiro (UFRJ), Rio de Janeiro, RJ Brazil; 6 Laboratório Misto Internacional “Sentinela”, FIOCRUZ, IRD, Universidade de Brasília (UnB), Rio de Janeiro, RJ Brazil

**Keywords:** Mosquitoes identification, MALDI-TOF MS, Optimization, Standardization

## Abstract

**Background:**

In the last decade, an innovative approach has emerged for arthropod identification based on matrix-assisted laser desorption ionization time-of-flight mass spectrometry (MALDI-TOF MS). Increasing interest in applying the original technique for arthropod identification has led to the development of a variety of procedures for sample preparation and selection of body parts, among others. However, the absence of a consensual strategy hampers direct inter-study comparisons. Moreover, these different procedures are confusing to new users. Establishing optimized procedures and standardized protocols for mosquito identification by MALDI-TOF MS is therefore a necessity, and would notably enable the sharing of reference MS databases. Here, we assess the optimal conditions for mosquito identification using MALDI-TOF MS profiling.

**Methods:**

Three homogenization methods, two of which were manual and one automatic, were used on three distinct body parts (legs, thorax, head) of two mosquito laboratory strains, *Anopheles coluzzii* and *Aedes aegypti*, and the results evaluated. The reproducibility of MS profiles, identification rate with relevant scores and the suitability of procedures for high-throughput analyses were the main criteria for establishing optimized guidelines. Additionally, the consequences of blood-feeding and geographical origin were evaluated using both laboratory strains and field-collected mosquitoes.

**Results:**

Relevant score values for mosquito identification were obtained for all the three body parts assayed using MALDI-TOF MS profiling; however, the thorax and legs were the most suitable specimens, independently of homogenization method or species. Although the manual homogenization methods were associated with a high rate of identification on the three body parts, this homogenization mode is not adaptable to the processing of a large number of samples. Therefore, the automatic homogenization procedure was selected as the reference homogenization method. Blood-feeding status did not hamper the identification of mosquito species, despite the presence of MS peaks from original blood in the MS profiles of the three body parts tested from both species. Finally, a significant improvement in identification scores was obtained for field-collected specimens when MS spectra of species from the same geographical area were added to the database.

**Conclusion:**

The results of the current study establish guidelines for the selection of mosquito anatomic parts and modality of sample preparation (e.g. homogenization) for future specimen identification by MALDI-TOF MS profiling. These standardized operational protocols could be used as references for creating an international MS database.

**Supplementary Information:**

The online version contains supplementary material available at 10.1186/s13071-022-05361-0.

## Background

Mosquitoes, which are hematophagous dipterans belonging to the Culicidae family, are considered to be arthropods of major public health importance [1–3]. The Culicidae family encompasses about 3550 species and subspecies of 44 genera [4–6]. Among these, *Anopheles* spp., *Aedes* spp. and *Culex* spp. are the most important vectors due to their role in the transmission of a variety of pathogens, including parasites, viruses and bacteria [4, 5]. Although these mosquitoes are mainly distributed in tropical and subtropical areas [6], global warming, together with the (long-distance) travel of people and transportation of goods, has facilitated the colonization of new areas. Such mosquito invasions has led to the emergence of diseases where they were previously non-existent, culminating in recurrent outbreaks and pandemics.

Mosquito-borne diseases (MBDs) are a worldwide concern [7, 8]. Identification of those mosquito species, among the wide diversity of mosquito species worldwide, involved in the transmission of MBD is of prime importance for vector surveillance and control programs [5, 9]. Morphological identification keys are the most widely used technique for entomological surveillance [10, 11]. Although this laborious approach remains the primary and key reference method for species identification, it requires robust entomological knowledge. A recent study revealed that only 81% and 64% of the entomologists participating in the study succeeded in identifying mosquito specimens at the genus and species level, respectively [12]. Furthermore, this technique is limited when specimens are damaged, belong to species complexes or are new in a specific area [13]. In recent years, molecular biology tools have emerged as important methods able to overcome these issues [12]. Molecular techniques target genes for species identification, such as the mitochondrial cytochrome* c* oxidase subunit 1 (COI), the internal transcribed spacer 2 (ITS2), the intergenic spacer (IGS) or regions from ribosomal subunits [14]. However, molecular methods are usually time-consuming and can be expensive, limiting their use in large-scale studies [15].

In this context, a relatively inexpensive methodology that is also rapid, technically reproducible and straightforward, and which allows large-scale processing, has emerged as an alternative, namely matrix-assisted laser desorption ionization time-of-flight mass spectrometry (MALDI-TOF MS) [15]. This proteomic tool, routinely used in microbiology diagnosis laboratories for the identification of bacteria or yeast [16–18], has been efficiently applied in several medical entomology studies for the identification of arthropods, such as flies [19, 20], ticks [21, 22] or fleas [23]. This tool was also successfully used to identify mosquitoes of laboratory or field origin [24–26]. Its performance regarding mosquito identification was highlighted in a recent study reporting the successful distinction of members from the *Anopheles gambiae* complex, *An. gambiae*, *An. coluzzii* and *An. arabiensis* field-collected [27].

Mosquito legs were the main body part initially used for specimen identification by MALDI-TOF MS [23, 28]. An optimized procedure was subsequently established to standardize MS identification of mosquitoes for sample preparation of adult and larval stages using legs or whole specimens, respectively [29]. The performance of imago mosquito identification by MS using legs has been repeatedly confirmed [26, 27, 30, 31]. However, mosquito legs have the disadvantage of being breakable, subsequently hampering MS identification of specimens that have lost their legs. To circumvent this limitation, the thorax was recently proposed as a complementary body part for mosquito identification by MS profiling [32]. MS profiling of these two body parts corroborated species identification by other means, reinforcing identification confidence and success rate [32, 33].

The increasing interest in mosquito identification by MALDI-TOF MS has led to the emergence of a wide diversity of procedures, including those for mode of sample preparation and selection of body part [15]. Regarding body parts, some studies reported the use of thoraxes and cephalothoraxes [34, 35]. More recently, Nabet et al. [24] emphasized that the mosquito head appears to be the most appropriate body part for MS identification. In addition, methods for homogenizing the samples vary according to the studies, with some studies using automatic devices for homogenization, such as the TissueLyser LT (TL; Qiagen, Hilden, Germany), and others using a micropette (MP) [24] or pellet pestles (PP) [36] to homogenize the samples manually. The sample homogenization mode and quantity of mix buffer could also influence the quality of MS spectra, altering the spectral matching with reference MS spectra from the database [32, 37]. Other factors, such as geographical origin [30, 37, 38] or engorgement status [24], have also been reported to induce variations in the MS spectra, which could affect species identification. The absence of a consensual strategy for mosquito identification using MALDI-TOF MS bias direct inter-study comparisons, limit the sharing of reference MS spectra databases (DB) and confuse new users.

It is therefore necessary to clarify the best mosquito body part for MS identification, the best procedures for sample preparation and the effect of some endogenous and exogenous factors on these MS profiles. In this context, we have compared the intra-species reproducibility and inter-species specificity of MS spectra from heads, thoraxes (without wings) and legs, homogenized either with automatic or manual modes, with the aim to determine the most suitable conditions for MS mosquito specimen identification. The impacts of mosquito blood engorgement and the geographical origin of specimens on MSPs were also assessed. Laboratory-reared and field-collected specimens from the same species were used for these evaluations.

## Methods

### Ethics statement

The study was conducted under the ethical clearance No. 2018/06/1036/CE/CNERSH/SP and No. 1284/CRERSHC/2021 granted by the Cameroon National (CNE) and Centre Regional (CRE) Ethics Committee for Research on Human Health. Authorization to carry out the study was obtained from the administration and heads of household (HoH) through an informed consent form. The volunteer collectors were adults living in the collection sites. After each collection performed per human landing catch (HLC), malaria prophylaxis was given to volunteer collectors. Mosquitoes from the Congo and other localities were collected at larval stages or with traps with no need for ethical authorizations. Mosquitoes from Cameroon and the Congo were shipped to VITROME (Vecteurs—Infections Tropicales et Mediterranéenne, Marseille, France) according to importation authorization No. ER-22-2020 and were provided by the Research Institute of Yaoundé. Eggs of mosquitoes from Brazil were kindly provided by the Oswaldo Cruz Foundation, according to the material transfer agreement and importation authorization No. ER-12-2018.

### Mosquitoes

Laboratory-reared and field-collected mosquitoes were used in this study (Table 1). The four laboratory strains used were: *Aedes aegypti* (Bora), originated from French Polynesia (i.e., Bora Bora); *Ae. albopictus* (Mrs), originated from the south of France (i.e. Marseille); *Anopheles coluzzii* (Dkr), originated from Senegal (i.e. Dakar); and *An. gambiae* (Kis), originated from Kenya (i.e. Kisumu). *Ae. aegypti* (Bora). *Aedes albopictus* (Mrs) and *An. coluzzii* (Dkr) were reared at VITROME, whereas *An. gambiae* (Kis) were reared at the IRY (Institut de Recherche de Yaoundé, Yaoundé, Cameroon). Breeding was performed under controlled conditions of temperature (28 ± 1 °C), relative humidity (80 ± 10%) and photoperiod (12/12-h light/dark) in an incubator (Panasonic cooled incubator) as previously described [37]. Briefly, eggs were laid to hatch in trays containing dechlorinated water and larvae were fed with fish feed (JBL Novo Prawn [JBL GmbH & Co. KG, Neuhofen, Germany] or Tetramin®[Tetra Werke, Melle, Germany]). Pupae were transferred to mosquito cages until the emergence of adults. Adults were fed with a 10% glucose solution. For egg production, blood meals were provided through a Parafilm membrane (Hemotek Membrane Feeding Systems; Hemoteck Ltd., Blackburn, UK) using fresh heparinized human blood [37]. Only female imago mosquitoes were included in the study. The mosquitoes were stored at − 20 °C until future analyses.

**Table 1:** Overview of mosquito origins, subgroups for database creation and parameters assessed

Species	Strains^a^	Country	Source	Stored	Number (engorged)^b^	DB1^c^	DB2^c^	DB3^c^	Parameters assessed^d^
*Aedes aegypti*	Bora Bora (Bora)	French Polynesia	Laboratory	Fresh	155 (110)	6^e^	6^e^	6^e^	A, B, C
*Ae. aegypti*	Oiapoque (Oia)	Brazil	Field	Frozen (- 20 °C)	15			2^f^	C
*Ae. albopictus*	Marseille (Mrs)	France	Laboratory	Fresh	15		2^f^	2^f^	C
*Ae. albopictus*	Dschang (Dsg)	Cameroon	Field	Frozen (− 20 °C)	15			2^f^	C
*Anopheles coluzzii*	Dakar (Dkr)	Senegal	Laboratory	Fresh	155 (110)	6^e^	6^e^	6^e^	A, B, C
*An. gambie* s.s.	Kisumu (Kis)	Kenya	Laboratory	Frozen (− 20 °C)	20		2^f^	2^f^	C
*An. coluzzii*	Tibati (Tib)	Cameroon	Field	Silicate, room temperature	16			2^f^	C
*An. gambie* s.s.	Brazaville (Bra)	Congo	Field	Silicate, room temperature	17			2^f^	C
Total					408 (120)	12	16	24	

*DB* Database,* MP* micropipette,* PP* pellet pestle,* TL* TissueLyser,* RT* room temperature

^a^The abbreviation of the name of each strain is given in parentheses

^b^Number of engorged specimens is indicated in parentheses

^c^Databases 1, 2, 3: Number of specimens included in the databases per body part and homogenization mode

**Fig. 1:**
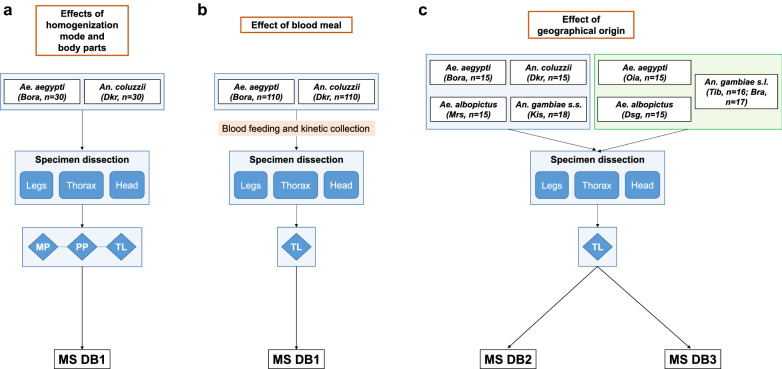
Experimental design of study. The different parameters evaluated were: **a** the homogenization procedures per body part, **b** the consequences of engorgement per body part, **c** the effect of mosquito origin on mosquito identification per body part. The mosquito species, their geographical origin and the number of specimens tested are indicated. The homogenization mode used is indicated in the diamond-shaped box. See the Table 1 for details on DB1, DB2 and DB3 and the origins of the mosquito species. Abbreviations: MP, Micropipette; PP, pellet pestle; TL, TissueLyser; MS DB, Mass spectrometry database

^d^Classification of parameters assessed according to Fig. 1. A: Effects of homogenization mode and body parts. B: Effect of blood meal. C: Effect of geographical origin

^e^Two specimens per body part homogenized either by MP, PP or TL mode

^f^Two specimens per body part homogenized by TL mode

Field collection of mosquitoes included larval and adult specimens from *Ae. albopictus* (Cameroon) and *An. gambiae* (Cameroon and Congo). Laboratory-reared, uniquely adult females were selected for this study. Mosquito details are presented in Table 1. Collected specimens were stored at − 20 °C or in silicate at room temperature from a few months to 1 year. All field-collected specimens were identified morphologically under a binocular loupe (Leica M80; Leica Microsystemes SAS, Nanterre, France) using morphological descriptions [9, 30]. Eggs from *Ae. aegypti* (Oia) originated from Brazil (i.e. Oiapoque) were hatched at the VITROME laboratory and raised until adulthood. Females were dissected (see Additional file 1: Data file) and stored at − 20 °C until their use.

### Molecular identification of field-collected mosquitoes

DNA was extracted using the QIAamp DNA tissue extraction kit (Qiagen), according to the manufacturer’s instructions. The molecular identification of mosquitoes was done as previously described [14, 39, 40] (see Additional file 1: Data file, for details).

### Preliminary tests for homogenization of adult mosquito

Volumes of 20, 30 and 40 µl, respectively, of a mix buffer consisting of a mix (50/50) of 70% (v/v) formic acid (Sigma-Aldrich Chimie, Lyon, France) and 50% (v/v) acetonitrile (Fluka, Buchs, Switzerland) were added to five individual heads of female mosquitoes per species, namely *Ae. aegypti* (Bora) and *An. coluzzii* (Dkr). The samples were homogenized using the automatic method described in the following section, prior to the MALDI-TOF MS study. Theintensity and reproducibility of the MSPs were the criteria used to establish the optimal volume of the mix buffer to use for mosquito head homogenization.

### Sample homogenization for MALDI-TOF MS analysis

Adults of laboratory strains *Ae. aegypti* (Bora) and *An. coluzzii* (Dkr) were used to compare the three homogenization procedures: manual homogenization using a MP or PP, and automatic homogenization using using the TL.

The heads, legs and thoraxes from 10 mosquitoes per species were used for each homogenization condition. The quantity of mix buffer added for homogenization was dependent on the body part analyzed. Accordingly, 30 and 50 µl of mix buffer were used for the legs and thoraxes, respectively, as previously described [32, 33], and 30 µl of mix buffer was used for the heads based on our preliminary tests.

For the manual crushing procedures, the samples were ground either with a MP (tip size: 10 µl) or PP, until complete homogenization had been achieved, as previously described [23, 24]. Automatic homogenization consisted of adding glass beads (diameter: 0.1 mm; BioSpec Products, Bartlesville, OK, USA) into each sample tube, followed by three homogenization cycles, each 1 min long, at 30 Hertz using the TL according to the standardized, automated setting described previously [29]. After sample homogenization, a quick spin-down centrifugation at 200 *g* for 1 min was performed, and 1 µl of the supernatant was loaded, in quadruplicate, into the MALDI-TOF MS steel target plate (Bruker Daltonics, Wissembourg, France). The grinding time for five samples was recorded per homogenization method and body part to estimate the time required per method.

### Mosquito engorgement

The two laboratory strains, *Ae. aegypti* (Bora) and *An. coluzzii* (Dkr), were used to assess the consequences of engorgement on the MSPs of the heads, legs and thoraxes. Mosquitoes were engorged with human blood provided through an Hemoteck artificial feeding membrane feeding system (Discovery Workshops, Accrington, UK), as described previously [41]. Engorged specimens were transferred to a new cage, and females were collected after 2, 6, 12, 24, 48 and 72 h post engorgement. Twenty females were collected per time point and species, with the exception of at 72 h, for which only 10 mosquitoes were assessed. The specimens were stored at − 20 °C until processing. For MALDI-TOF MS analysis, each specimen was dissected, and the heads, legs and thoraxes were homogenized using the automated TL protocol described above.

### Sample loading on MALDI-TOF MS target plate

A 1-μl aliquot of the supernatant of each sample was spotted on the MALDI-TOF MS steel target plate (Bruker Daltonics) in quadruplicate and covered with 1 μl of matrix solution (saturated α-cyano-4-hydroxycinnamic acid [Sigma-Aldrich Chemie]), 50% (v/v) acetonitrile, 2.5% (v/v) trifluoroacetic acid (Sigma-Aldrich Chemie), following which HPLC-grade water was added. The sample was then analyzed on a Microflex LT MALDI-TOF Mass Spectrometer device (Bruker Daltonics). Details on sample loading, MALDI-TOF MS parameters and MS spectra analysis are given in Additional file 1: Data file.

### Creation of reference databases and blind tests

Three databases were created for this study to assess the impact of body part selection, sample homogenization mode, engorgement or geographical origin of specimens on the accuracy of species identification (Fig. 1; Table 1). The reference MS spectra were created using spectra from the heads, legs and thoraxes of two specimens per species, as shown in Table 1, using MALDI-Biotyper software v3.0. (Bruker Daltonics) [38]. All specimens included in the databases were not engorged. MS spectra were created with an unbiased algorithm using information on peak position, intensity and frequency. The first database (DB1) comprised MS spectra of the heads, legs and thoraxes from two laboratory strains, *Ae. aegypti* (Bora) and *An. coluzzii* (Dkr), homogenized either by the MP, PP or TL method. This reference MS DB was used to assess the effect of homogenization mode per body part (Fig. 1a) and the impact of blood meal on MS spectra (Fig. 1b). DB2 and DB3 were composed of MS spectra on the heads, legs and thoraxes from laboratory-reared or laboratory-reared plus those from field-collected mosquito species, respectively (see Table 1 for details), with the aim to assess the impact of geographical origin on specimen identification.

A total of 24 unfed and 110 engorged specimens per *Ae. aegypti* (Bora) and *An. coluzzii* (Dkr) species were tested against DB1, whereas 124 and 116 specimens from four distinct mosquito species, including laboratory-reared and field-collected mosquitoes, were used against the DB2 and DB3. The reliability of species identification was estimated using the log score values (LSVs). This score, which ranged from 0 to 3, was calculated using a biostatistical algorithm from the MALDI Biotyper software v.3.0. According to previous studies [33, 42, 43], LSVs > 1.8 can be considered to be reliable for species identification. Data were analyzed using R software (R core Team; R Foundation for Statistical Computing, Vienna, Austria).

### Statistical analysis

After verifying that the LSVs in each group (homogenization mode, body parts) did not follow a Gaussian distribution (Shapiro–Wilk test), the Kruskal–Wallis and Mann–Whitney tests were computed when appropriate using R software (R core Team; R Foundation for Statistical Computing). Frequencies were compared by the Chi-square test. All differences were considered significant at *P* < 0.05.

## Results

### Quantity of mix buffer appropriate for homogenization of mosquito head and MS analysis

Five adult *Ae. aegypti* (Bora) and five *An. coluzzii* (Dkr) mosquitoes were used to determine the appropriate quantity of mix buffer to add to the mosquito head for protein extraction before sample homogenization with the TL device and MALDI-TOF MS analysis. The visual comparison of MS spectra according to the volume of mix buffer used indicated a high similarity per species (Additional file 3: Figure S1A, B). The mean composite correlation index (CCI) values of the mosquito head MS spectra were elevated, ranging from 0.77 to 0.84, and were not significantly different per species, independent of which volume of mix buffer was used (Kruskal–Wallis test, *P* > 0.05) (Additional file 3: Figure S1C). The analysis of the MSPs and CCI values indicated a good reproducibility of head MS spectra independently of the mix buffer volume used for sample homogenization. It is interesting to note that a slight decrease in MSP intensity was noted for both species testd when 40 µl of mix buffer was used (Additional file 3: Figure S1A-B). We concluded that 20 or 30 µl of mix buffer was the most suitable and, therfore, to limit the number of experimental variables, we chose the volume of 30 µl, as used for homogenization of the legs, for head homogenization.

### Consequences of homogenization procedures and mosquito body part on MSPs

**Fig. 2:**
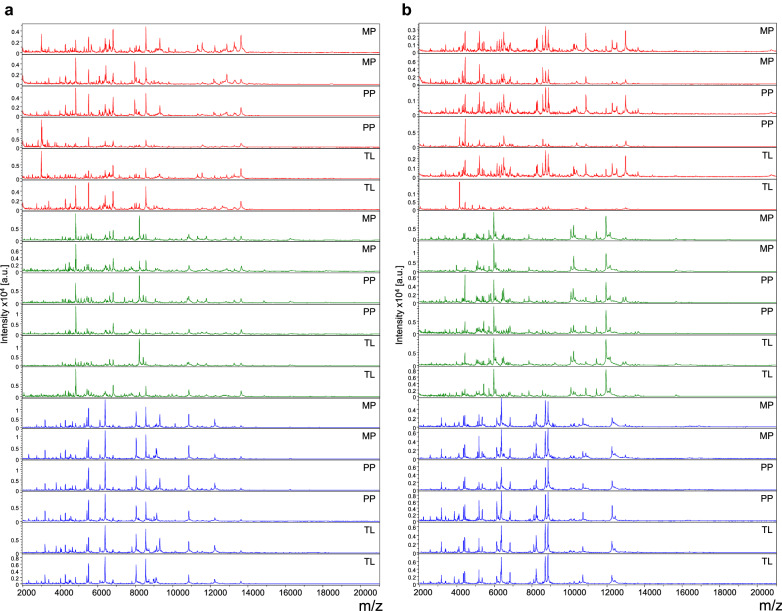
Comparison of MALDI-TOF MS spectra from heads, legs and thoraxes of *Aedes aegypti* (Bora) and *Anopheles coluzzii* (Dkr) homogenized using distinct methods. Representative MS spectra from heads (red), legs (green) and thoraxes (blue) of *Ae. aegypti* (Bora) (**a**) and *An. coluzzii* (Dkr) (**b**) are shown. MS spectra from two distinct specimens per body part and homogenization method were obtained using FlexAnalysis v3.4 software. Abbreviations: a.u., Arbitrary units; MALDI-TOF MS, matrix-assisted laser desorption/ionization-time-of-flight mass spectrometry; m/z, mass-to-charge ratio

**Fig. 3:**
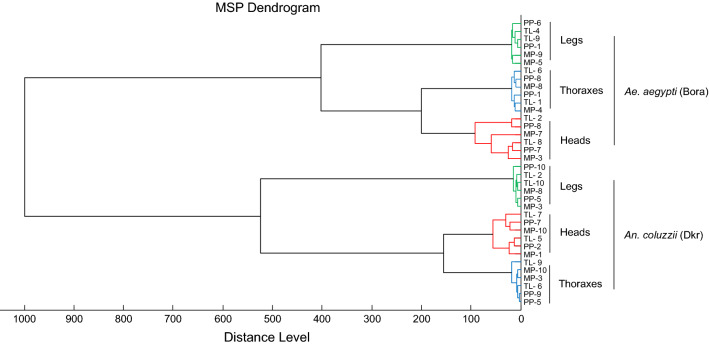
MSP dendrogram of MALDI-TOF MS spectra from heads (red), legs (green) and thoraxes (blue) of *Ae. aegypti* (Bora) and *An. coluzzii* (Dkr) homogenized using distinct methods. Diagram is based on two specimens per species, per body part and per homogenization method. The distance units correspond to the relative similarity of MS spectra. The dendrogram was created by Biotyper v3.0 software. Abbreviations: MSP, Main spectra profile

Heads, thoraxes (without wing) and legs from adult *Ae. aegypti* (Bora) and *An. coluzzii* (Dkr) were homogenized using either the TL, MP or PP method prior to analysis by MALDI-TOF MS. The three body parts, from 10 specimens per species, were tested per homogenization mode. A total of 180 samples generated 720 high-intensity MS spectra, independently of the mosquito body part and homogenization method. The MSPs were visually reproducible per body part for each species (Fig. 2). Interestingly, the MS patterns appeared to be both species- and body part-specific. Cluster analysis using two specimens per species and per homogenization method revealed that all samples from the same mosquito species clustered on the same branch (Fig. 3). Samples were grouped per body part for each species, reflecting spectra reproducibility. For each body part, the intertwining of spectra, independently of the homogenization mode, emphasized that the homogenization method did not appear to impact MS spectra. The results of this cluster analysis suggest that the primary determinant for the MSPs is the species, followed by the body part, with MS spectra for the legs being distinctive compared to those of the heads and thoraxes.

**Fig. 4:**
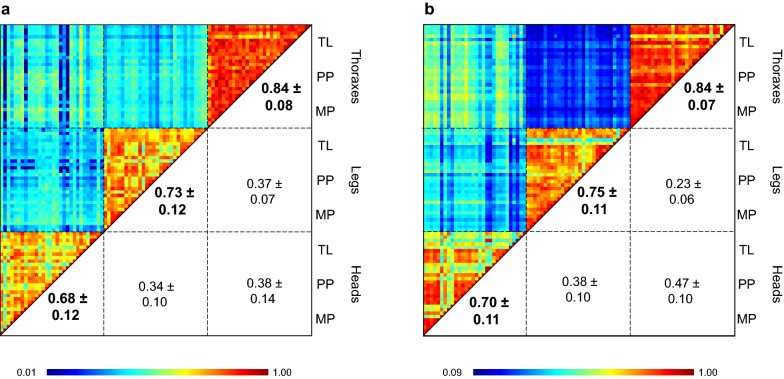
CCI matrix value representing the levels of MS spectra reproducibility between heads, legs and thoraxes of *Ae. aegypti* (Bora) (**a**) and *An. coluzzii* (Dkr) (**b**) homogenized with distinct modes. The levels of MS spectra reproducibility are indicated in red and blue, revealing relatedness and incongruence between spectra, respectively. The values correspond to the mean coefficient correlation and respective standard deviations obtained for paired condition comparisons. Numbers in bold correspond to CCI values obtained for each body part independently of the homogenization mode used. CCI was calculated with MALDI-Biotyper v3.0 software. CCI, Composite Correlation Index

A CCI-based analysis confirmed the reproducibility of MS spectra per body part and per species independently of the homogenization mode (Fig. 4). Effectively, the mean CCI values of each body part were comparable across homogenization modes for both species. However, for both species, the mean CCI values obtained for thoraxes were higher than those for legs, followed by heads. The comparisons of mean CCI values showed significant differences between thoraxes and legs (Mann–Whitney test, *P* < 0.0001), thoraxes and heads (Mann–Whitney test, *P* < 0.0001) and legs and heads (Mann–Whitney test, *P* < 0.042) from *Ae. aegypti* (Bora). Similarly, significant differences in mean CCI values were obtained for *An. coluzzii* (Dkr), between thoraxes and legs (Mann–Whitney test, *P* < 0.0001), thoraxes and heads (Mann–Whitney test, *P* < 0.0001) and legs and heads (Mann–Whitney test, *P* < 0.009). These results underline a decrease in MS spectra reproducibility in the order thoraxes to legs and heads. The low mean CCI values obtained for pairwise comparisons of MS spectra from two distinct body parts for both species, ranging from 0.23 ± 0.06 (mean ± standard deviation) to 0.47 ± 0.10, confirmed that these MSPs are body part specific (Fig. 4).

### Efficiency of mosquito identification according to body part and homogenization modes by MS

**Fig. 5:**
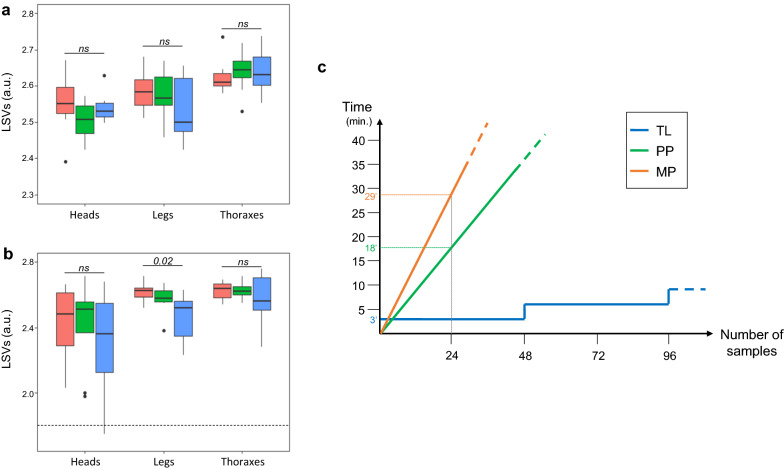
Evaluation of the performance of each homogenization mode taking into account sample identification accuracy and duration of sample preparation. LSVs from MS spectra of heads, legs and thoraxes of *Ae. aegypti* (Bora) (**a**) and *An. coluzzii* (Dkr) (**b**), using distinct homogenization methods, were compared. Dashed line represents the threshold values (LSV ≥ 1.8) for relevant identification. Significant differences between homogenization modes per body part are indicated (Kruskal–Wallis test). **c** Duration of sample preparation according to homogenization method. This graph is based on average data measured and estimated using legs, heads and thoraxes from five mosquito samples homogenized either with a MP (orange), PP (green) or TL (blue) (details in Additional file 8: Table S1). The time required for processing 24 samples according to homogenization mode are presented. Abbreviations: LSV, Log score value; ns, not significant

The MS spectra used for MSP dendrogram analysis were included as reference MS spectra to create DB1 (Table 1; Additional file 2: Data file). Then, each body part (legs, thoraxes and heads) from eight adult specimens per species [*Ae. aegypti* (Bora) and *An. coluzzii* (Dkr)] homogenized by MP, PP or TL, corresponding to a total of 144 samples, were submitted, in quadruplicate to MALDI-TOF MS analysis and queried against DB1 (Fig. 1a). All samples were correctly classified at the species and body part levels (Figs. 5a, b). With the exception of the MS spectra from three *An. coluzzii* (Dkr) head samples, highly relevant identification scores were obtained (LSVs ≥ 2.0), independently of the homogenization mode used. The higher LSVs indicated the high quality and reproducibility of the obtained MS spectra.

To assess the performances of MALDI-TOF MS for mosquito identification according to homogenization mode, LSVs were compared for each body part and species (Fig. 5a, b). No significant difference (Kruskal-Wallis test, *P* > 0.05) was noted between the homogenization modes per body part, with the exception for legs from *An. coluzzii* (Dkr) (Kruskal-Wallis test, *P* = 0.02; Fig. 5b). Although LSVs from the legs of *An. coluzzii* (Dkr) obtained with the automatic mode (TL) were significantly lower than those obtained from the MP mode (Mann-Whitney test, (*P* = 0.01), identification scores remained highly relevant (LSVs > 2.2), thereby preventing misidentification risk. LSVs from the legs of *An. coluzzii* (Dkr) did not differ between homogenization modes.

The comparison of the LSVs per homogenization mode, independently of the body part, revealed no significant differences (Kruskal-Wallis test, *P* > 0.05) for both species (Additional file 4: Figure S2A–B). Conversely, significantly different LSVs were obtained among body parts for *Ae. aegypti* (Bora) (Kruskal-Wallis test, *P* < 0.001) and *An. coluzzii* (Dkr) (Kruskal-Wallis test, *P* = 0.028), independently of the homogenization mode used (Additional file 4: Figure. S2C-D). Paired comparisons revealed a significant better matching against DB1 of MS spectra of thoraxes compared to MS spectra of legs (Mann-Whitney test, *P* < 0.01) or to MS spectra of heads (Mann-Whitney test, *P* < 0.001) from *Ae. aegypti* (Bora). For *An. coluzzii* (Dkr), LSVs were also significantly higher in thoraxes than in heads (Mann-Whitney test, *P* = 0.023). These results indicate that higher LSVs were obtained with MS spectra of thoraxes followed, in decreasing LSV, by those of the legs and heads, confirming the data obtained on the MSP dendrogram or from the CCI analyses.

### Duration of sample processing according to body part and homogenization mode

To determine which homogenization method is the more advantageous, the time required for sample processing for each method was measured and estimated for larger specimen collections, as complementary criteria. Then, heads, legs and thoraxes from five *Ae. aegypti* (Bora) were ground with MP, PP or TL by two of the authors and processing duration was recorded. For both manual modes, sample homogenizations were quickest for the heads and thoraxes, followed by the legs (Additional file 8: Table S1). Among the manual grinding modes, the PP method was less time-consuming than than the MP mode by 1.5-fold. However, when the number of samples to process was very low (i.e. < 5), the automatic sample homogenization mode with TL was generally more rapid than both manual methods, independently of body part. From one to 48 samples could be processed using the TL in only 3 min, whereas in this time period between one and 36 samples could be processed using PP, or up to 58 min was needed for the same number of samples using MP (Fig. 5c). TL was then the faster method for sample homogenization, independently of researcher, the number of samples to process or the body part selected. Based on these results, we concluded that the automatic procedure (i.e. TL) seemed to be the more appropriate method for sample homogenization and this method was used for the successive experiments.

### Consequence of mosquito blood meal on MSPs according to body parts

To assess whether mosquito blood-feeding status could affect MSPs and subsequent mosquito identification, adult females of *Ae. aegypti* (Bora) and *An. coluzzii* (Dkr) were collected kinetically at 2, 6, 12, 24, 48 and 72 h post-engorgement, and their heads, thoraxes and legs were analy\ed by MALDI-TOF MS (Fig. 1b). MS spectra from heads, thoraxes and legs of not engorged *Ae. aegypti* (Bora) and *An. coluzzii* (Dkr) specimens, as well as MS spectra from human blood provided for mosquito meals were used as the control for MS profile comparisons (Additional file 5: Fig. S3).

High-intensity MS spectra were obtained for 20 specimens per species and body part tested at each time point, with the exception of the 72 h post-blood-feeding time point when only 10 specimens were tested. The visual comparison of the 660 MS spectra using Flex Analysis v3.4 software revealed that, for the vast majority of the samples (> 80% of samples), there was no apparent change compared to respective body part and species from unfed specimens (Additional file 9: Table S2). In the samples in which MS profile changes were observed, these modifications corresponded to the appearance of MS peaks at about 7568 m/z and 15,138 m/z (Additional file 5: Figure S3). These two MS peaks, also present in MS profiles from human blood, were considered to be blood contaminants of the mosquito MS spectra. These foreign MS peaks were found in all body parts and at 2–48 h post-feeding. Interestingly, the intensity of peaks corresponding to human blood signature decreased with the increasing delay post-blood feeding (Additional file 5: Figure S3). This observation is likely attributable to the digestion process of the blood meal. However, this blood signature was more frequently found in the thorax samples (Additional file 9: Table S2).

### Identification of engorged mosquitoes by MS

**Fig. 6:**
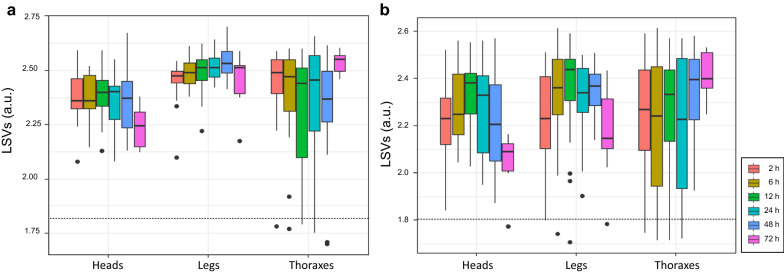
Consequences of blood engorgement on mosquito identification per body part. LSVs from MS spectra of heads, legs and thoraxes of *Ae. aegypti* (Bora) (**a**) and *An. coluzzii* (Dkr) (**b**), kinetically collected after human blood engorgement, were compared. Collection time points are distinguished by color code, as indicated in the right panel. Dashed line represents the threshold values (LSV ≥ 1.8), for relevant identification. All samples were homogenized using TL mode

To assess the consequences of blood engorgement on the identification of mosquitoes, MS spectra from the 660 samples were queried against DB1 (Fig. 1b). The proportion of correct and relevant (LSVs ≥ 1.8) identifications reached 96.5% (*n* = 637/660) for MS spectra from both species independently of the body part and length of delay post-feeding (Fig. 6). Among the 23 samples identified as being without relevant LSVs (i.e. < 1.8), 11 were from thoraxes of *Ae. aegypti* (Bora) and 12 from *An. coluzzii* (Dkr) distributed in heads (*n* = 1), legs (*n* = 3) and thoraxes (*n* = 8). The detection of MS peaks of a blood origin was visible in half of these (*n* = 12/23), all from thorax samples.

The comparison of LSVs between MS spectra with and without peaks for foreign blood for each mosquito species revealed a significant decrease in matching scores (Mann–Whitney test, *P* < 0.001) only for MS spectra from *Ae. aegyPti* (Bora) (Additional file 6: Figure S4A–B). Nevertheless, the proportion of correct and relevant (LSVs ≥ 1.8) identifications for mosquito MS spectra with or without peaks of foreign blood remained high, reaching 90.6% (*n* = 116/128) and 97.9% (*n* = 521/532), respectively. Regarding mosquito body parts, the MS spectra of thoraxes of *Ae. aegypti* (Bora) and *An. coluzzii* (Dkr) had significantly lower LSVs (Mann–Whitney test, *P* < 0.001; Additional file 6: Figure. S4C and Mann–Whitney test, *P* < 0.004, Additional file 6: Figure S4D, respectively). Although MS peaks for foreign blood seemed to more affect thoraxes’ match scoring, correct and relevant LSVs (> 1.8) were obtained for the large majority of MS spectra for thoraxes human blood MS peaks (80.3%, *n* = 49/61).

### Impact of mosquito origin on the identification and LSV distribution

**Fig.7:**
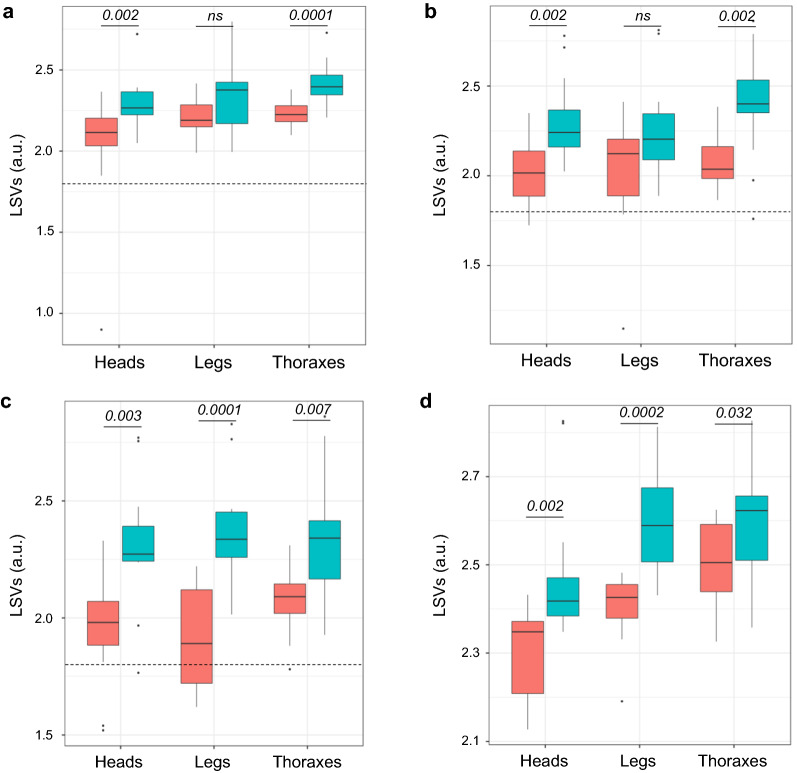
Effect of geographical origin on mosquito identification per body part. LSVs from MS spectra of field-collected mosquito species: **a** *An. gambiae s.s.* (Bra), **b** *An. coluzzii* (Tib), **c** *Ae. albopictus* (Dsg),** d** *Ae. aegypti* (Oia) against DB2 (pink) and DB3 (blue). Significant differences of LSVs obtained between DB2 and DB3 per species and body part are indicated (Mann–Whitney test). Dashed line represents the threshold values (LSV ≥ 1.8) for relevant identification. All samples were homogenized using TL mode. Abbreviation: ns, Not significant

To assess whether MS spectra variations occurred for specimens from the same species but from distinct geographical origins, MS spectra from foour distinct mosquito species, laboratory-reared or field-collected, were queried against DB2 and DB3. The median LSVs against DB2 were 2.21, 2.32 and 2.36 for heads, legs and thoraxes, respectively, regardless of the species analyzed. The distribution of LSVs varied significantly between body parts (Kruskal-Wallis test, *P* = 0.002), with the lowest scores for heads (Additional file 7: Figure S5). The proportion of correct and relevant (LSVs > 1.8) identifications against DB2 ranged from 79.0% for heads to 83.9% for legs (Table 2). The query of these MS spectra against the DB3, upgraded with MS spectra from field specimens, did not significantly improve the proportion of correct and relevant identifications (Chi-square tests, *P* > 0.05). Conversely, LSVs obtained per body part, per field species, were significantly improved between DB2 and DB3 for nearly all paired comparisons ( Mann–Whitney test, *P* < 0.05; Fig. 7). Misidentifications concerned mainly MS spectra from *An. gambiae* sensu lato, underlining the difficulty in classifying specimens from a species complex.

**Table 2:** MALDI-TOF MS identification results of species from different origins after crosswise testing against DB2 and DB3

Query against databases^a^	Species	Origin^b^	Number^c^	Correct species, *n* (%)^d^			Incorrect species, *n* (%)^d^		
				Heads	Thoraxes	Legs	Heads	Thoraxes	Legs
BD2	*Ae. aegypti*^e^	French Polynesia (Bora)	15	15 (100)	15 (100)	15 (100)	0 (0.0)	0 (0.0)	0 (0.0)
	*Ae. aegypti*	Brazil (Oia)	15	15 (100)	15 (100)	15 (100)	0 (0.0)	0 (0.0)	0 (0.0)
	*Ae. albopictus*^e^	France (Mrs)	13	13 (100)	13 (100)	13 (100)	0 (0.0)	0 (0.0)	0 (0.0)
	*Ae. albopictus*	Cameroon (Dsg)	15	15 (100)	15 (100)	13 (86.7)	0 (0.0)	0 (0.0)	2 (13.3)
	*An. coluzzi*^e^	Senegal (Dkr)	15	5 (33.3)	12 (80.0)	15 (100)	10 (66.7)	3 (20.0)	0 (0.0)
	*An. gambiae* s.s.^e^	Kenya (Kis)	18	18 (100)	18 (100)	18 (100)	0 (0.0)	0 (0.0)	0 (0.0)
	*An. coluzzii*	Cameroon (Tib)	16	4 (25.0)	0 (0.0)	4 (25.0)	12 (75.0)	16 (100)	12 (75.0)
	*An. coluzzii*	Congo (Bra)	3	2 (66.7)	0 (0.0)	0 (0.0)	1 (33.3)	3 (100)	3 (100)
	*An. gambiae* s.s.^e^	Congo (Bra)	14	11 (78.6)	14 (100)	11 (78.6)	3 (21.4)	0 (0)	3 (21.4)
Total (%)			124	98 (79.0)	102 (82.3)	104 (83.9)	26 (21.0)	22 (17.7)	20 (14.5)
BD3	*Ae. aegypti*^e^	French Polynesia (Bora)	15	15 (100)	15 (100)	15 (100)	0 (0.0)	0 (0.0)	0 (0.0)
	*Ae. aegypti*	Brazil (Oia)	13	13 (100)	13 (100)	13 (100)	0 (0.0)	0 (0.0)	0 (0.0)
	*Ae. albopictus*^e^	France (Mrs)	13	13 (100)	13 (100)	13 (100)	0 (0.0)	0 (0.0)	0 (0.0)
	*Ae. albopictus*	Cameroon (Dsg)	13	13 (100)	13 (100)	13 (100)	0 (0.0)	0 (0.0)	0 (0.0)
	*An. coluzzi*^e^	Senegal (Dkr)	15	5 (33.3)	13 (86.7)	15 (100)	10 (66.7)	2 (13.3)	0 (0.0)
	*An. gambiae* s.s.^e^	Kenya (Kis)	18	18 (100)	18 (100)	18 (100)	0 (0.0)	0 (0.0)	0 (0.0)
	*An. coluzzii*	Cameroon (Tib)	14	10 (71.4)	6 (42.9)	5 (35.7)	4 (28.6)	8 (57.1)	9 (64.3)
	*An. coluzzii*	Congo (Bra)	3	2 (66.7)	0 (0.0)	1 (33.3)	1 (33.3)	3 (100)	2 (66.7)
	*An. gambiae* s.s.^e^	Congo (Bra)	12	4 (25.0)	11 (91.7)	5 (41.66)	8 (75.0)	1 (8.3)	7 (58.3)
Total (%)			116	93 (80.2)	102 (87.9)	98 (84.5)	23 (19.8)	14 (12.1)	18 (15.5)

*s.s.* sensu stricto

^a^See Table 1 for details on DB2 and DB3

^b^Country of origin of the sample (abbreviation for the name of each strain is given in parentheses)

^c^Number of samples blindly tested against databases (DB2 or DB3)

^d^*n*: number of samples with correct (same name as determined by molecular methods) or incorrect (different name from that determined with molecular methods) species identification. %: prevalence or proportion of samples

^e^Mosquito strains were laboratory reared

## Discussion

MALDI-TOF MS profiling has revolutionized clinical microbiology in the context of microorganism identification [44, 45], and the versatility and robustness of this method, as well as practical aspects, has resulted in modernization of the approaches to arthropod monitoring during the last decade [15]. In addition to its success in identifying specimens from more than a dozen arthropod families, including Culicidae [15, 46], this innovative proteomic tool has been used pragmatically to detect pathogenic agents in vectors [47–49] or determine the trophic preferences of such vectors [41, 50]. The main limitation to its wide usage at the present time is the absence of a public reference MS spectra DB containing arthropod spectra formally certified after reliable morphological and molecular identification. Nevertheless, prior to creating and sharing reference MS spectra DBs, it is essential to establish a standardized protocol. The absence of consensus procedures for sample preparation have contributed in past studies to a heterogeneity of results, hampering the comparison and sharing of MS spectra [24, 43, 48]. The present study assessed a number of intrinsic parameters, such as body part selected from the mosquito for MS study or geographical origin of mosquito, but also extrinsic factors, such as blood-feeding status or the mode of sample homogenization. All of these factors, among others, may moderately to markedly affect the resulting MS spectra [24, 43, 48].

Although mosquito legs have been the body part most frequently used for specimen identification by MS analysis [26, 27, 30, 31], for which a standardized protocol has been established [29, 43], other anatomical parts, including thoraxes [32, 33], cephalothoraxes [34, 35] or heads [24], have also been studied by MS. These latter body parts were chosen, notably, to prevent the risk of non-identification from specimens having lost all their breakable legs. In the present study, our analysis of MS spectra of three body parts from two mosquito species using CCI revealed that the highest reproducibility of protein profiles was obtained for thoraxes, followed by legs and heads. The lower branch distances obtained by cluster analysis for legs and thoraxes compared to heads for both species underlined a more important heterogeneity of MS profiles for this last body part. Moreover, the significant higher LSVs obtained for thoraxes compared to legs and heads confirmed that thoraxes appear to be the most appropriate body part for mosquito identification by MS profiling. Nevertheless, the high proportions (90%) of correct and relevant identifications (LSVs > 1.8) obtained for legs and heads support the use oft both body parts.

Conversely to our findings, a recent work pointed out that mosquito heads generated MS spectra with the highest reproducibility compared to legs and thoraxes [24]. Such inconsistent results highlight the necessity to propose guidelines for mosquito species identification by MS profiling. This divergence in results could be attributed to numerous factors that impact the quality of MS spectra, such as the storage conditions of field-collected specimens and the protocol applied [29, 36]. Nabet et al. performed sample homogenization using a unique volume of mix buffer independently of the body part [24], whereas in the present study we selected the most appropriate volume of mix buffer based on our experiments with heads or on previous studies on legs and thoraxes [28, 32]. Using an inadequate volume of mix buffer could reduce protein extraction and could likely explain, at least in part, the heterogeneity of MS profiles per body part [37].

The homogenization mode of samples has also been reported to impact the quality of MS spectra [29]. In the present study, we noted no significant differences in LSVs between the two manual methods (MP and PP)and one automatic method (TL), independently of the body part and species tested. The high reproducibility obtained with both manual modes was likely attributed to the low number of samples treated by experienced researchers. The performance of manual homogenization methods is probably inversely proportional to the number of samples to process and directly linked to the skills of those performing the experiments. Moreover, manual homogenization becomes a bottleneck in the pipeline of mosquito identification due to the amount of time required for this step. Effectively, the estimated time required to process 24 samples was, respectively, six- to ten-fold longer using PP and MP compared to TL mode. As MALDI-TOF MS is well-adapted for high-throughput analyses, applying an automatic homogenization procedure that limits variations in sample handling and enables a large number of samples to be tested appears to be the more appropriate procedure. The establishment of an automatized procedure for mosquito larvae homogenization [29, 37] allowed the application of MALDI-TOF MS to monitor mosquito fauna at immature stages for several months [51, 52]. The homogenization step was automatized to reduce improper sample grinding and improve the acquisition of high-quality MS spectra. Currently, no quality control step to reject outlier MS spectra is available in commercial software (e.g. MALDI-Biotyper v3.0. from Bruker Daltonics). Some R packages have bee developed to examine MS spectra quality [53, 54], but they require a minimum of computational knowledge. In the future, the systematic application of a quality control step of MS spectra prior to their query against MS reference DB will allow for filtering of atypical spectra and then prevent inaccurate identification.

To improve mosquito identification, notably for sibling or cryptic species, one proposal has been to to submit, independently, two distinct body parts from the same specimen for MS analysis. This strategy was applied to the thoraxes and legs of mosquitoes, and the results corroborated the identification obtained per body part and enhanced the identification confidence level [32, 33]. The same strategy applied to ticks permitted the classification of unambiguously closely related *Ixodes* species [21]. There are advantages to testing more than one body part. First, in the case of a damaged specimen, at least one of the body parts selected remains intact for MS submission. Second, for cryptic species, the double DB query with distinct compartments could confirm identification in cases of doubt. Interestingly, in the present study, cluster analysis showed that thorax and head MS spectra were on closer branches to each other than were leg MS spectra to the thorax and head MS spectra, for both species. This close vicinity reflects the proximity of the thorax and head MS profiles. The risk of cross-matching of MS spectra between thoraxes and heads of the same species is therefore more probable and has already been reported [24]. Thus, when two body parts from the same specimen are used to improve and corroborate mosquito species identification, it appears more judicious to pair legs and thoraxes or legs and heads rather than the couple heads and thoraxes.

The impairment of arthropod identification by MS when freshly blood-engorged specimens are used has been reported in earlier investigations [19, 55]. In sand flies, this failure of specimen identification was attributed to the potential presence of blood traces in the thoraxes impacting MS patterns by masking species-specific biomarker masses [55]. Conversely, other researchers assessing the performances of MALDI-TOF MS for determining host blood origin from engorged sand flies using abdomens successfully identified these field-collected specimens by submitting respective thoraxes to MS [56]. In the present study, we noted no drastic change in the MS profile for engorged mosquitoes from both species, independently of the body part and delay in post-feeding, compared to unfed specimens. More than 95% of the samples were correctly and relevantly identified. A blood signature was detected in only 19.4% of the samples tested from engorged specimens, represented by mainly two MS peaks that presumably correspond to the mono- (15,138 m/z) and double-charge (7568 m/z) of the same kind of protein. This 15-kDa MS peak has been observed in engorged sand flies [57] and mosquitoes [41].

The inconstant detection of the blood MS peaks in engorged specimens, the detection of the same blood MS peaks in MS spectra from the three body parts and the inverse correlation of blood peak intensity with the delay of blood-feeding support the hypothesis that the origin of blood signatures come likely from contamination which occurred during dissection of engorged specimens rather than from remaining host blood. It is quite possible that blood leakage could occur during the separation of the thorax from the abdomen of freshly engorged specimens, thus compromising the cleanness of the other body parts, as has been reported in other arthropods [56, 57]. Nevertheless, as the amount of blood that stained the other body parts is generally low, MS specimen identification remains generally possible [27, 57]. The authors of one study reported a decrease in the identification rate of MS from the thoraxes of blood-fed mosquitoes [24]. In the same study, MS spectra from thoraxes of unfed mosquitoes were also less efficient for specimen identification compared to legs or heads. It is then likely that other factors, such as the sample preparation protocol, could explain the lower reproducibility of mosquito thorax MS spectra [24]. To limit blood contamination, the engorged specimens could be frozen and dissected onto a refrigerated plate to prevent sample thawing and, subsequently, blood leak.

MS profile variations according to the geographical origin has been reported for mosquitoes at the adult [24, 35, 38] or immature stages [37], but also other for families, such as sand flies [58] or ticks [21, 22, 59]. However, these MS spectra variations generally did not hamper the correct identification. In the present study, the addition of MS spectra from field specimens did not significantly improve the proportion of correct and relevant identification for the three body parts. Nevertheless, the upgrading of the DB significantly increased the LSVs from field specimens. The results of the present study confirm that some variations in MS spectra occurred due to the geographical origin of specimens from the same species, and these were observed for the three body parts. However, other factors, such as the storage conditions [29] or duration of storing [43], could also contribute to intra-species MS profile variations. Therefore, we conclude that MS spectra of specimens from the same species collected in different areas remain sufficiently close for their correct and relevant identification, despite the absence of MS spectra of specimens from all locations in the DB. Nevertheless, as these MS spectra improve the score of identification confidence, the introduction of MS spectra from local specimens is recommended. Complementary experiments with mosquitoes from the same species with multiple geographical origins, such as the invasive *Ae. albopictus* species, remain necessary to confirm these data. Moreover, in parallel to the creation of an international reference MS spectra DB for mosquito identification, the control of the inter-laboratory reproducibility of species-specific MS spectra using the same body part and standardized protocol becomes compulsory.

## Conclusions

**Fig. 8:**
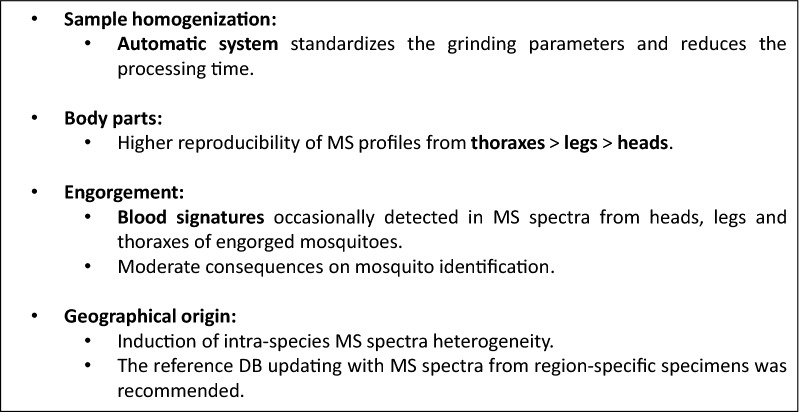
Summary of the main results and guidelines proposed for MS identification of adult mosquitoes

The interest in MALDI-TOF MS profiling is notably attributable to its advantages over molecular methods and morphological identification, with low costs of reagents and fast and straightforward sample preparation and data analyses, all of which do not require specialized expertise. As several factors can affect MS spectra and, consequently, species classification, the challenge to new users hoping to apply the MALDI-TOF MS tool for mosquito identification is to navigate the complexity of these factors. The main outcomes and guidelines for MS identification of adult mosquitoes are summarized in Fig. 8. The homogenization of samples using automatic systems appears to be more appropriate, notably for the large number of samples that can be handled, standardization of the homogenization parameters and reduction of processing time. We found that heads, legs and thoraxes were effective material for mosquito identification. However, the higher reproducibility of MS profiles from thoraxes, followed in decreasing reproducibility, by legs and finally heads, evidenced the distinct level of performance according to body part. Moreover, the high diversity of legs and thorax MS spectra from the same specimen could improve species identification rate and level of confidence upon independent MS submission. Blood signatures could be detected in MS spectra from the heads, legs and thoraxes of engorged mosquitoes, and these signatures infrequently impaired mosquito identification. As geographical origin induces heterogeneity in MS spectra from specimens of the same species, implementing reference DBs with MS spectra from region-specific specimens would circumvent this limitation. Finally, the present work established guidelines for the selection of mosquito anatomic parts and the modality of sample preparation for future specimen identification by MALDI-TOF MS profiling. These protocols could be used as references for creating an international MS database.

## Supplementary Information

**Additional file 1.** Complementary details about the material and methods applied for mosquito dissection, molecular identification of field collected mosquitoes, sample loading on MALDI-TOF MS target plate, MALDI-TOF MS parameters and MS spectra analysis.

**Additional file 2.** Raw MS spectra from heads, legs and thoraxes of mosquitoes included in the MS reference database. MS spectra were obtained using a Microflex LT MALDI-TOF Mass Spectrometer (Bruker Daltonics). Details of each sample were listed of the excel file named “REF_MS_Spectra_Mosq_Body-parts_Januray-2022”.

**Additional file 3: Figure S1.** Quantity of mix buffer required for homogenization of mosquito heads. Representative head MS spectra of (**a**) *Aedes aegypti* (Bora) and (**b**) *Anopheles coluzzii *(Dkr) homogenized with 20 µl (**a**,** b**), 30 µl (**c**,** d**) or 40 µl (**e**,** f**) of mix buffer using TL mode. MS spectra from two distinct specimens per species and conditions were presented. a.u., arbitrary units; m/z, mass-to-charge ratio. (**c**) Composite correlation index (CCI) matrix representing the levels of MS spectra reproducibility between mosquito heads according to the mix buffer volume used. Results from five specimens per condition and species are presented. The levels of MS spectra reproducibility are indicated in red and blue, revealing relatedness and incongruence between spectra, respectively. CCI are expressed as the mean ± standard deviation.

**Additional file 4: Figure S2.** Comparison of LSVs from (**a**,** c**) *Ae. aegypti *(Bora) and (**b**,** d**) *An. coluzzii *(Dkr) according either to (**c**,** d**) body parts or (**a**,** b**) homogenization mode used. Significant differences of LSVs obtained between MS spectra were indicated in the right corner of each panel (Kruskal Wallis test). Mann-Whitney test were used for paired comparisons. Dashed line represents the threshold values (LSV ≥ 1.8), for relevant identification. a.u., arbitrary units; LSVs, log score values; m/z, mass-to-charge ratio; ns, not significant; MP, micropipette; PP, pellet pestle; TL, TissueLyser.

**Additional file 5: Figure S3.** Comparison of MS spectra from heads (**a**,** d**), legs (**b**,** e**) and thoraxes (**c**,** f**) of (**a**,** b**,** c0**) *Aedes aegypti* (Bora) and (**d**,** e**,** f**) *Anopheles coluzzii *(Dkr), after blood engorgement and kinetically collected. MS spectra with (orange) or without (light blue) MS peaks shared with human blood MS profiles are presented. Dotted square highlight these shared MS peaks. MS spectra from human blood (red) were used as reference on each panel. MS spectra from respective species and body part of unfed mosquitoes were presented on each panel (dark blue). Time point collection post blood feeding were indicated on each MS spectra. a.u., arbitrary units; m/z, mass-to-charge ratio; h, hours. * Blood peak was not found in samples from 72h.

**Additional file 6: Figure S4.** Comparison of LSVs from mosquito species with and without blood signature on their MS spectra*. *The LSVs obtained for (**a**,** c**) *Ae. aegypti* (Bora) and (**b**,** d**) *An. coluzzii *(Dkr) irrespective (**a**,** b**) or respective (**c, d**) to body parts classified according to the detection of MS peaks from blood origin were compared. Significant differences of LSVs obtained between MS spectra with (blue) and without (red) blood signature were indicated (Mann-Whitney test). Dashed line represents the threshold values (LSV ≥ 1.8), for relevant identification. All samples were homogenized using TL mode. a.u., arbitrary units; BS, blood signature; LSV, log score value; TL, TissueLyser.

Additional file 7**: Figure S5.** Comparison of LSVs between heads, legs and thoraxes, independently of the species, from laboratory or field origins. Significant differences of LSVs obtained between body parts were indicated in the right corner (Kruskal Wallis test). Dashed line represents the threshold values (LSV ≥ 1.8), for relevant identification. All samples were homogenized using TL mode. a.u., arbitrary units; LSV, log score value; TL, TissueLyser.

**Additional file 8: Table S1.** Time required for samples processing according to body part and homogenization mode.

**Additional file 9: Table S2.** Proportion and number of specimens with blood signature in the MS spectra

## Data Availability

The datasets of MS reference spectra added to the MS DB in the current study are freely available and downloadable from the Additional file 2.

## Abbreviations

CCI: Composite correlation index
LSVs: Log score values
MALDI-TOF MS: Matrix-assisted laser desorption/ionization-time-of-flight mass spectrometry
MBDs: Mosquito-borne diseases
MSP: Main Spectrum Profile
MP: Micropipette
PP: Pellet pestle
TL: TissueLyser
