# Supplementary material for: Enhanced procedures for mosquito identification by MALDI-TOF MS
Source: Parasit Vectors. 2022 Jun 30;15:240. doi: 10.1186/s13071-022-05361-0 (PMC9248115; doi:10.1186/s13071-022-05361-0)
Supplement: Supplementary file 1 — Additional file 1. Complementary details about the material and methods applied for mosquito dissection, molecular identification of field collected mosquitoes, sample loading on MALDI-TOF MS target plate, MALDI-TOF MS parameters and MS spectra analysis. [file 13071_2022_5361_MOESM1_ESM.docx]

**Additional file 1.** Complementary details about the material and methods applied for mosquito dissection, molecular identification of field collected mosquitoes, sample loading on MALDI-TOF MS target plate, MALDI-TOF MS parameters and MS spectra analysis.

***Mosquito dissection***

Legs, thoraxes without wings, and heads from each individual mosquito were dissected using sterile surgical blades (Swann-Morton, England) under a binocular loupe (Leica M80, Leica, Nanterre, France), transferred separately to a 1.5 mL Eppendorf tubes and stored at -20°C until MALDI-TOF MS analyses. The remaining body part (abdomen and wings) was used for molecular analyses, when required. A nomenclature was established to pair body parts from the same specimen.

***Molecular identification of field collected mosquitoes***

DNA was individually extracted from the abdomen of 2 mosquito specimens per species selected for MS reference database creation (n = 24) using the QIAamp DNA tissue extraction kit (Qiagen, Hilden, Germany), according to the manufacturer’s instructions. Molecular identification of mosquito at species level was performed by PCR product sequencing from a fragment of the cytochrome c oxidase 1 gene (cox1) using the primers LCO1490 (forward) (5'-GGT CAA CAA ATC ATA AAG ATA TTG G-3') and HC02198 (reverse) (5'-TAA ACT TCA GGG TGA CCA AAA AAT CA-3') as previously described [14]. The sequences were assembled and analysed using the ChromasPro software version 1.7.7 (Technelysium Pty. Ltd., Tewantin, Australia). All sequences were compared with sequences in the GenBank database using BLAST (http://blast.ncbi.nlm.nih.gov/Blast.cgi) and the Barcode of Life Data Systems (BOLD; http://www.barcodinglife.org) to assign unknown cox1 sequences to mosquito species. To distinct *An. gambiae s.s.* from *An. coluzzii*, the protocol developed by Scott et al [39] was applied, as previously described [40].

***Sample loading on MALDI-TOF MS target plate***

One microliter of supernatant of each sample was spotted on the MALDI-TOF MS steel target plate (Bruker Daltonics) in quadruplicate and air-dried at room temperature. Each spot was covered with 1 μL of matrix solution, composed of saturated α-cyano-4-hydroxycinnamic acid (Sigma-Aldrich), 50% (v/v) acetonitrile, 2.5% (v/v) trifluoroacetic acid (Sigma-Aldrich) prepared with HPLC-grade water**.** To control matrix quality (i.e. absence of MS peaks due to impurities), matrix solution was loaded in duplicate onto each MALDI-TOF MS plate alone. After air drying, the plate was placed in a Microflex LT MALDI-TOF Mass Spectrometer device (Bruker Daltonics) for analysis. For MS target plates loaded with field samples, heads, legs and thoraxes from two *Ae. aegypti* (Bora) specimens were included on each plate and were used as positive controls.

***MALDI-TOF MS parameters***

Protein mass profiles were obtained using a Microflex LT MALDI-TOF MS (Bruker Daltonics, Bremen, Germany), with detection in the linear positive-ion mode at a laser frequency of 50 Hz within a mass range of 2–20 kDa. The acceleration voltage was 20 kV, and the extraction delay time was 200 ns. Each spectrum corresponded to ions obtained from 240 laser shots performed in six regions of the same spot and automatically acquired using the AutoXecute method of the flexControl v2.4 software (Bruker Daltonics). The spectrum profiles were visualized with Flex analysis v.3.4 and exported to ClinProTools v2.2 and MALDI-Biotyper v3.0. (Bruker Daltonics, Germany) for data processing (smoothing, baseline subtraction, peak picking) and evaluation with cluster analysis. The detailed settings followed Nebbak et al. [36].

***MS spectra analysis***

MS spectra per homogenization mode and body part from *Ae. aegypti* Bora and *An. coluzzii* (Dkr) strains were compared to determine the most reproducible MS spectra. The reproducibility of spectra was evaluated using one or more of the following methods: visual comparison of the average spectra (MSP, Main Spectrum Profile) obtained from the four spectra of each sample performed by flexAnalysis v3.3 and ClinProTools v2.2 software (Bruker Daltonics), comparison of Log Score Values (LSVs) of MS queries against the homemade reference database using MALDI-Biotyper v3.0. software (Bruker Daltonics) and the use of the Composite Correlation Index (CCI) tool to assess spectra variations within and between each sample group according to parameters tested and body part, as previously described [36]. CCI was computed using the default settings of mass range 3000–12000 Da, resolution 4, 8 intervals and autocorrelation off. A CCI match value of 1 represents a perfect correlation, whereas a CCI match value of 0 represents an absence of correlation. To visualize the numerical values of CCI, a heat-map was automatically generated. Higher LSVs and correlation values, expressed by mean ± standard deviation (SD), reflected higher reproducibility of MS spectra and were used to determine the best homogenization conditions for automated sample preparation. These tools were also applied to compare MS spectra per body part, to assess the effect of engorgement on MS profiles and their relevance to identification, and also to evaluate spectra variations attributed to geographical origin.

**References**

14. Folmer O, Black M, Hoeh W, Lutz R, Vrijenhoek R. DNA primers for amplification of mitochondrial cytochrome c oxidase subunit I from diverse metazoan invertebrates. Mol Mar Biol Biotechnol. 1994;3:294–9.

39. Scott JA, Brogdon WG, Collins FH. Identification of Single Specimens of the Anopheles Gambiae Complex by the Polymerase Chain Reaction. Am J Trop Med Hyg. 1993;49:520–9.

40. Diabate A, Brengues C, Baldet T, Dabiré KR, Hougard JM, Akogbeto M, et al. The spread of the Leu-Phe kdr mutation through Anopheles gambiae complex in Burkina Faso: genetic introgression and de novo phenomena. Trop Med Int Health. 2004;9:1267–73.

36. Nebbak A, El Hamzaoui B, Berenger J-M, Bitam I, Raoult D, Almeras L, et al. Comparative analysis of storage conditions and homogenization methods for tick and flea species for identification by MALDI-TOF MS. Med Vet Entomol. 2017;31:438–48.
