# Supplementary figures and images for: Enhanced procedures for mosquito identification by MALDI-TOF MS
Source: Parasit Vectors. 2022 Jun 30;15:240. doi: 10.1186/s13071-022-05361-0 (PMC9248115; doi:10.1186/s13071-022-05361-0)

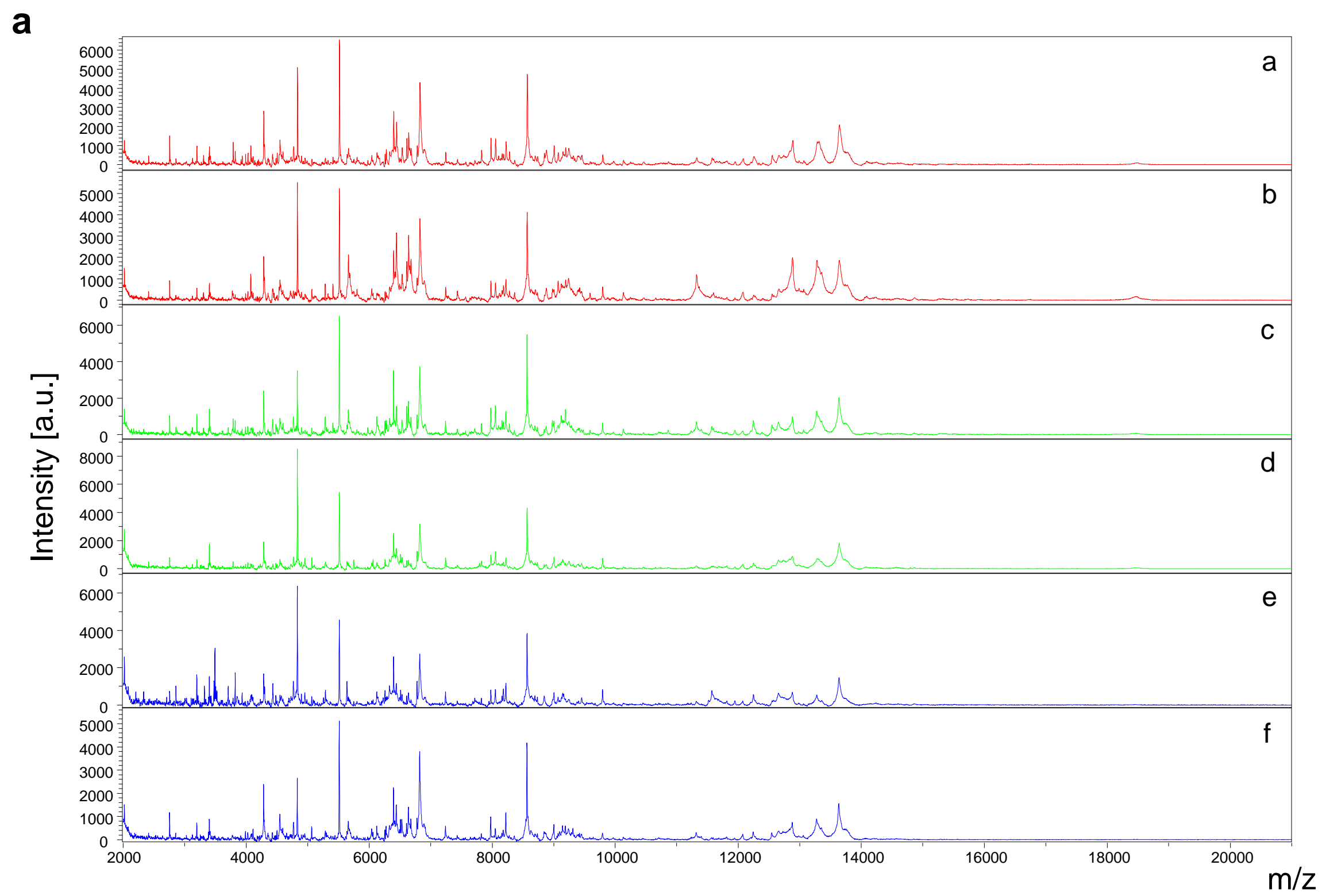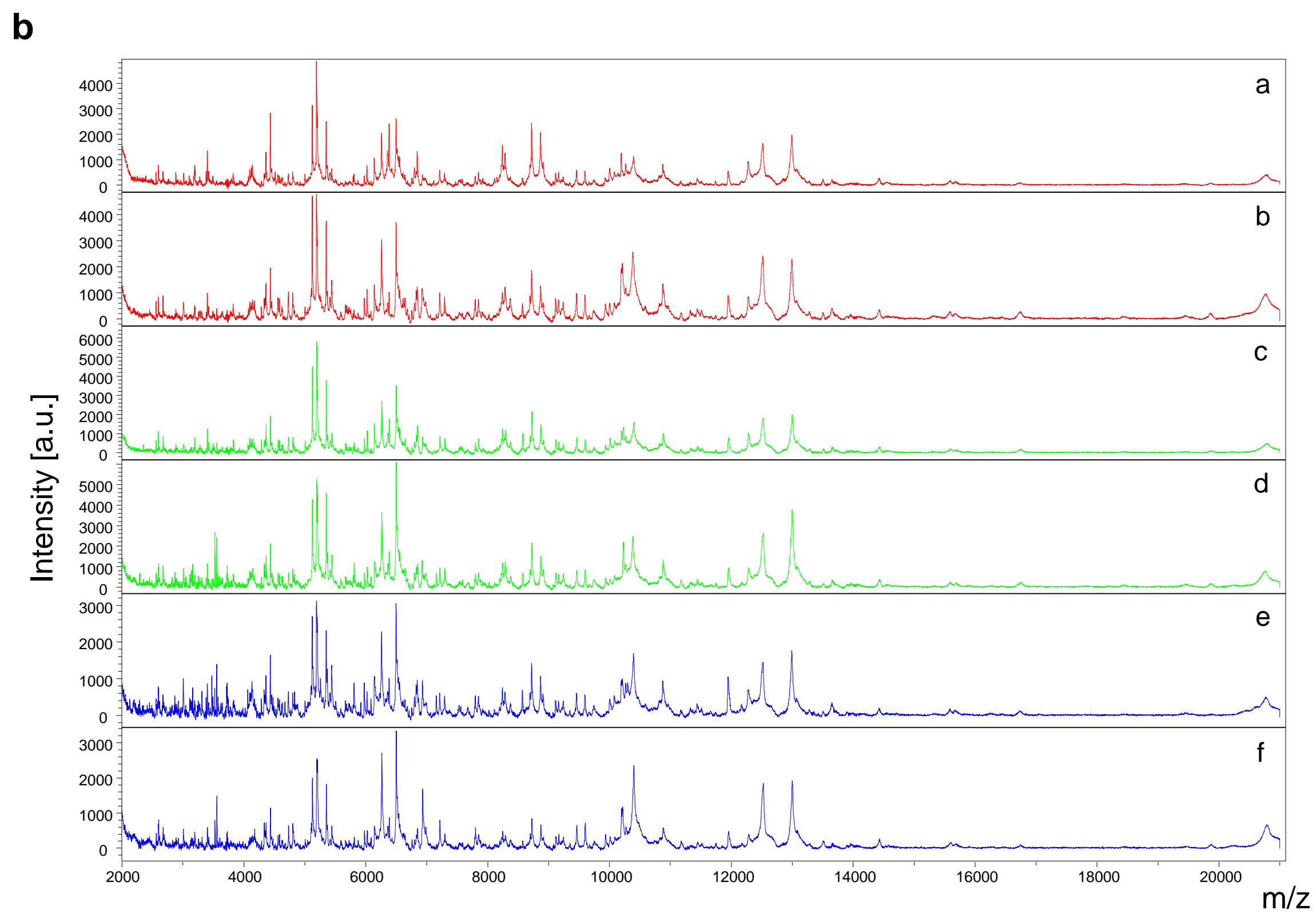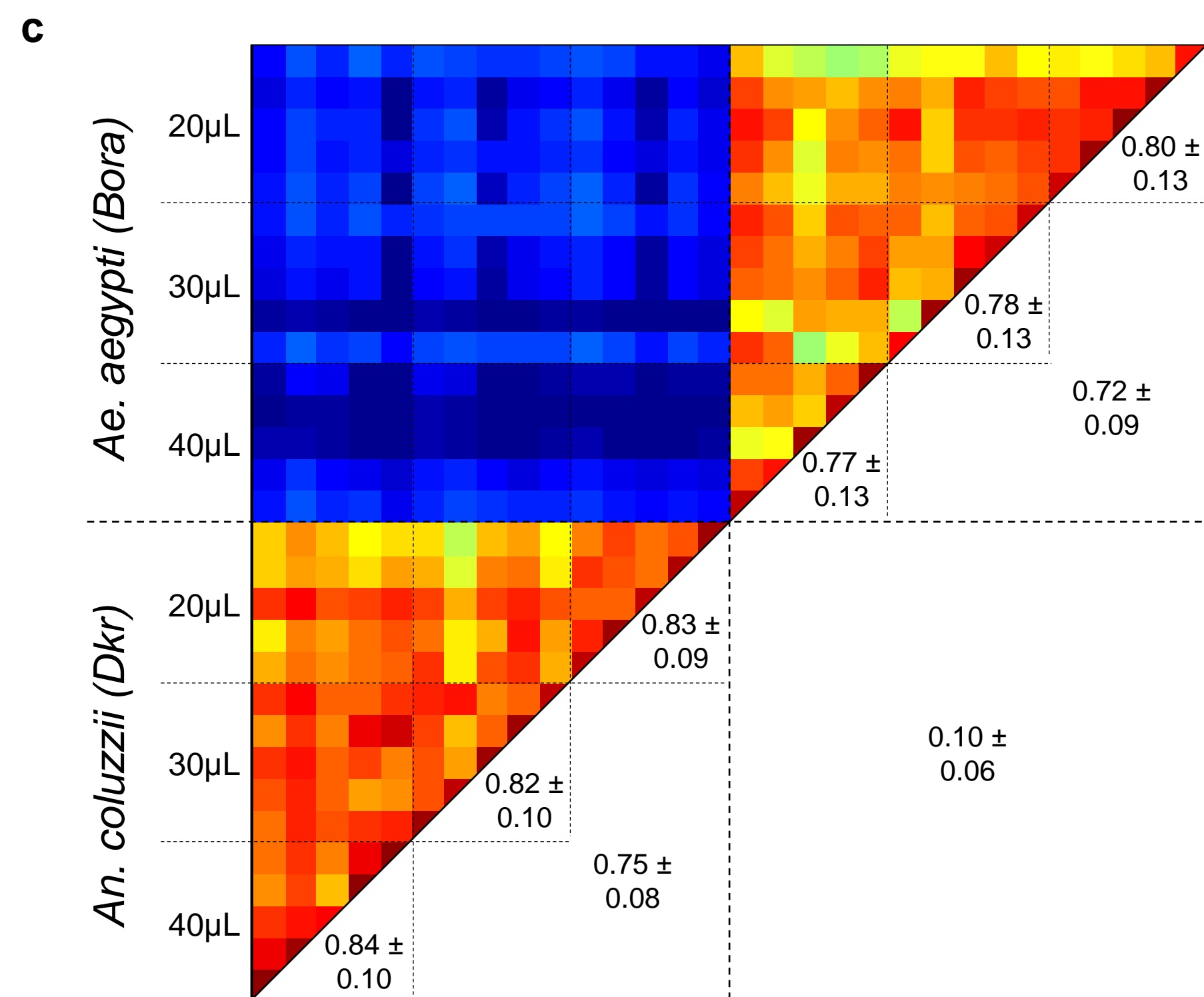

Supplement: Supplementary file 3 — Additional file 3: Figure S1. Quantity of mix buffer required for homogenization of mosquito heads. Representative head MS spectra of (a) Aedes aegypti (Bora) and (b) Anopheles coluzzii (Dkr) homogenized with 20 µl (a, b), 30 µl (c, d) or 40 µl (e, f) of mix buffer using TL mode. MS spectra from two distinct specimens per species and conditions were presented. a.u., arbitrary units; m/z, mass-to-charge ratio. (c) Composite correlation index (CCI) matrix representing the levels of MS spectra reproducibility between mosquito heads according to the mix buffer volume used. Results from five specimens per condition and species are presented. The levels of MS spectra reproducibility are indicated in red and blue, revealing relatedness and incongruence between spectra, respectively. CCI are expressed as the mean ± standard deviation. [file 13071_2022_5361_MOESM3_ESM.pdf]

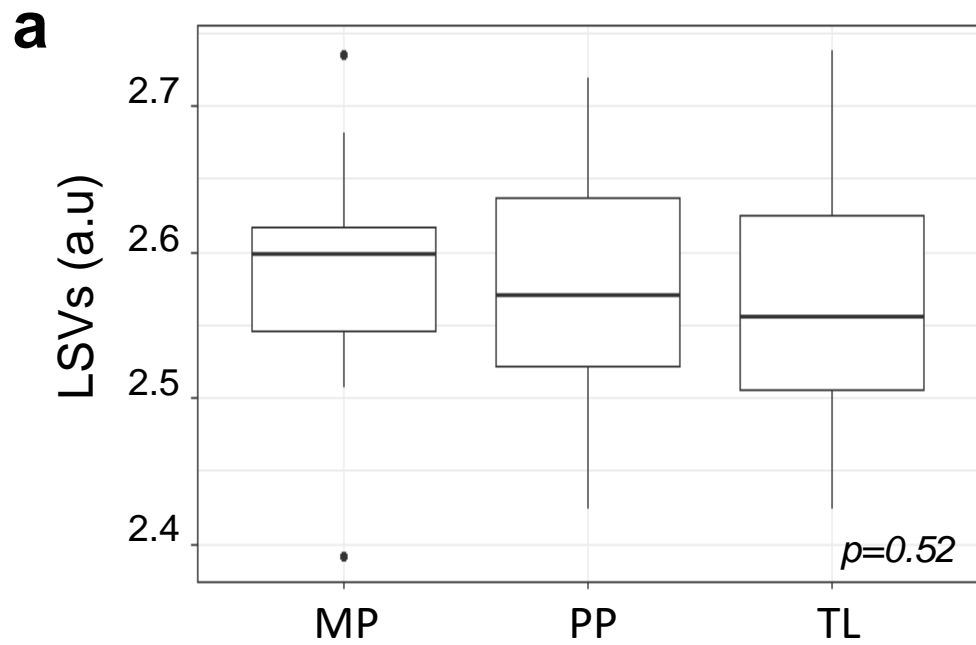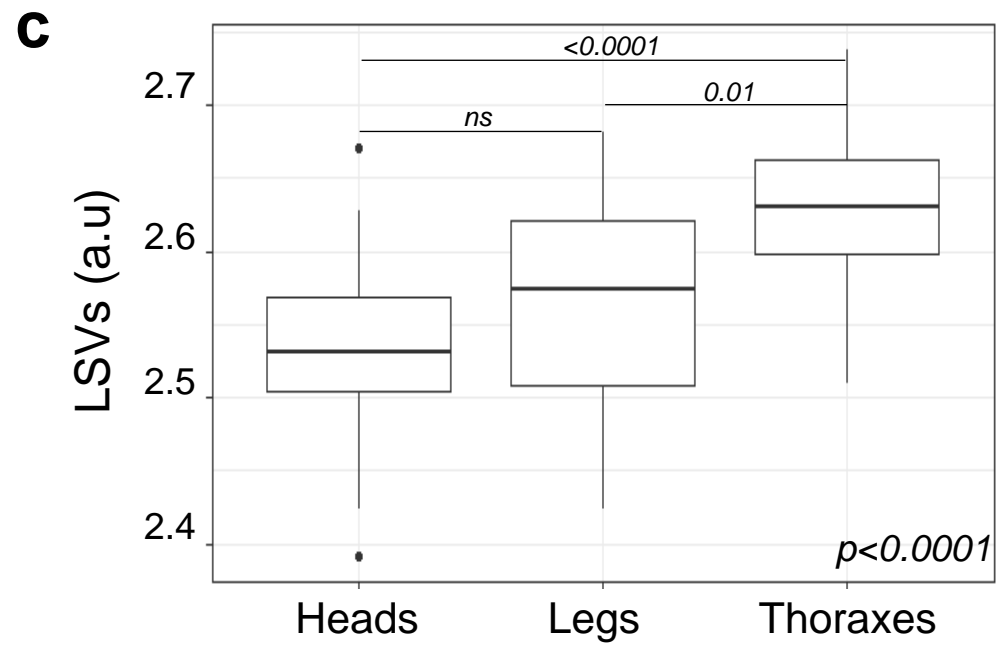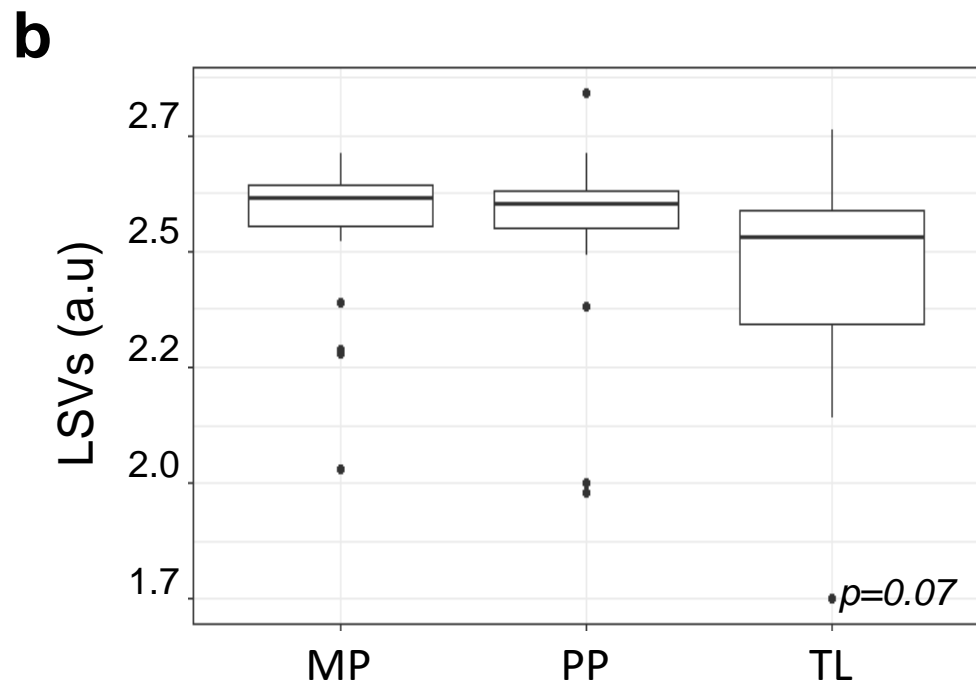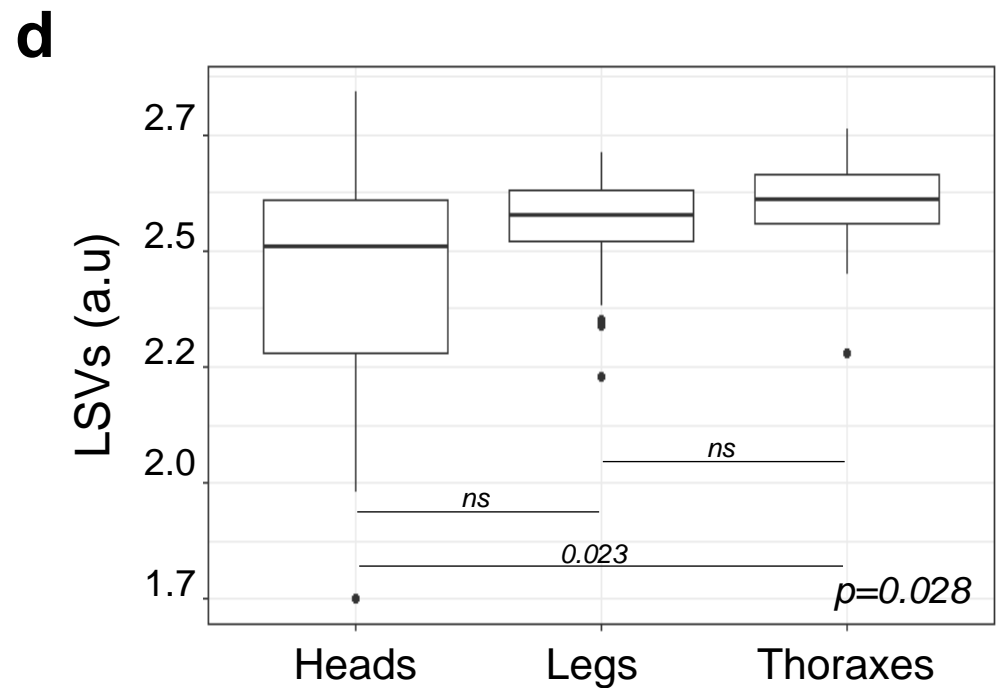

Supplement: Supplementary file 4 — Additional file 4: Figure S2. Comparison of LSVs from (a, c) Ae. aegypti (Bora) and (b, d) An. coluzzii (Dkr) according either to (c, d) body parts or (a, b) homogenization mode used. Significant differences of LSVs obtained between MS spectra were indicated in the right corner of each panel (Kruskal Wallis test). Mann-Whitney test were used for paired comparisons. Dashed line represents the threshold values (LSV ≥ 1.8), for relevant identification. a.u., arbitrary units; LSVs, log score values; m/z, mass-to-charge ratio; ns, not significant; MP, micropipette; PP, pellet pestle; TL, TissueLyser. [file 13071_2022_5361_MOESM4_ESM.pdf]

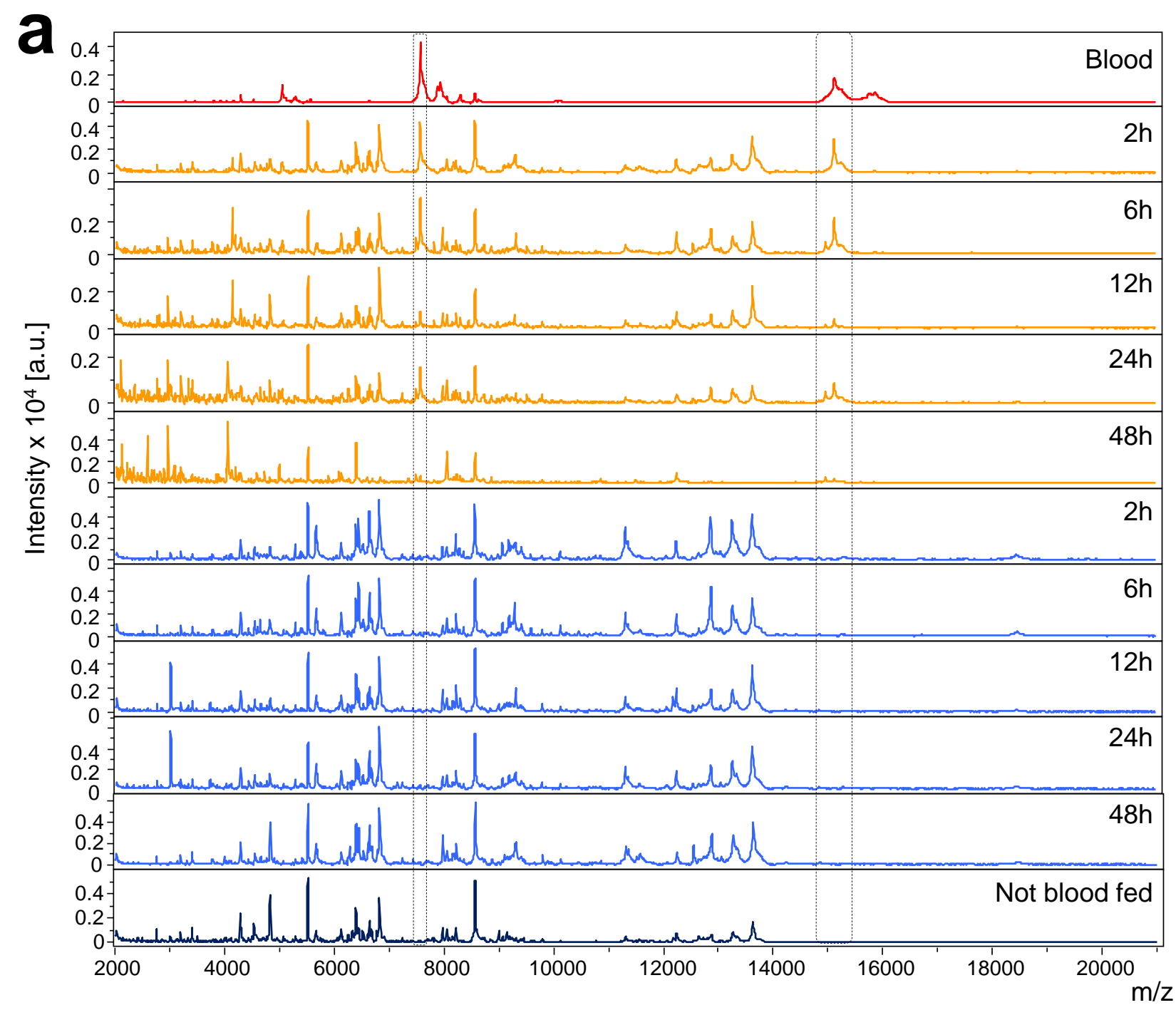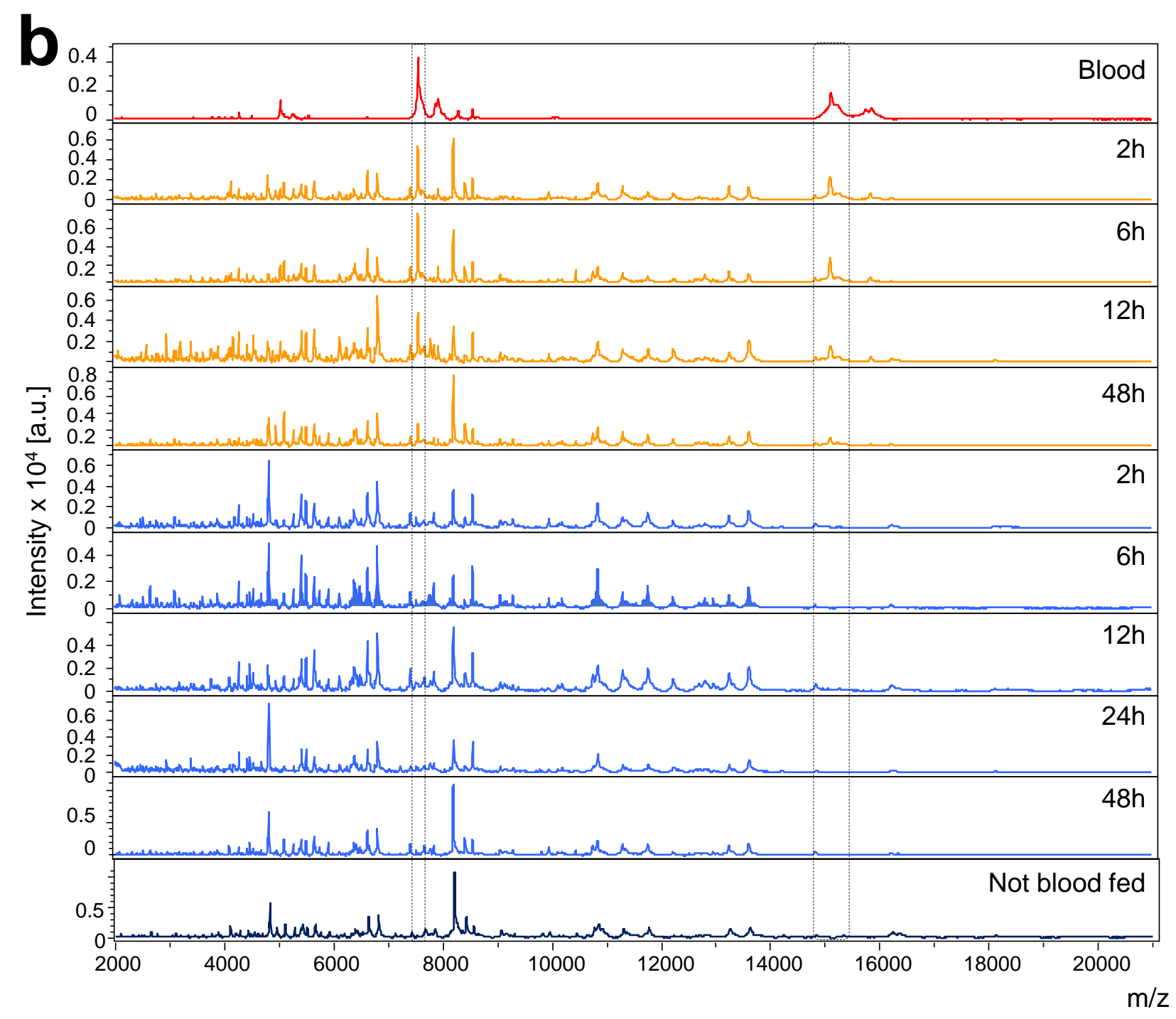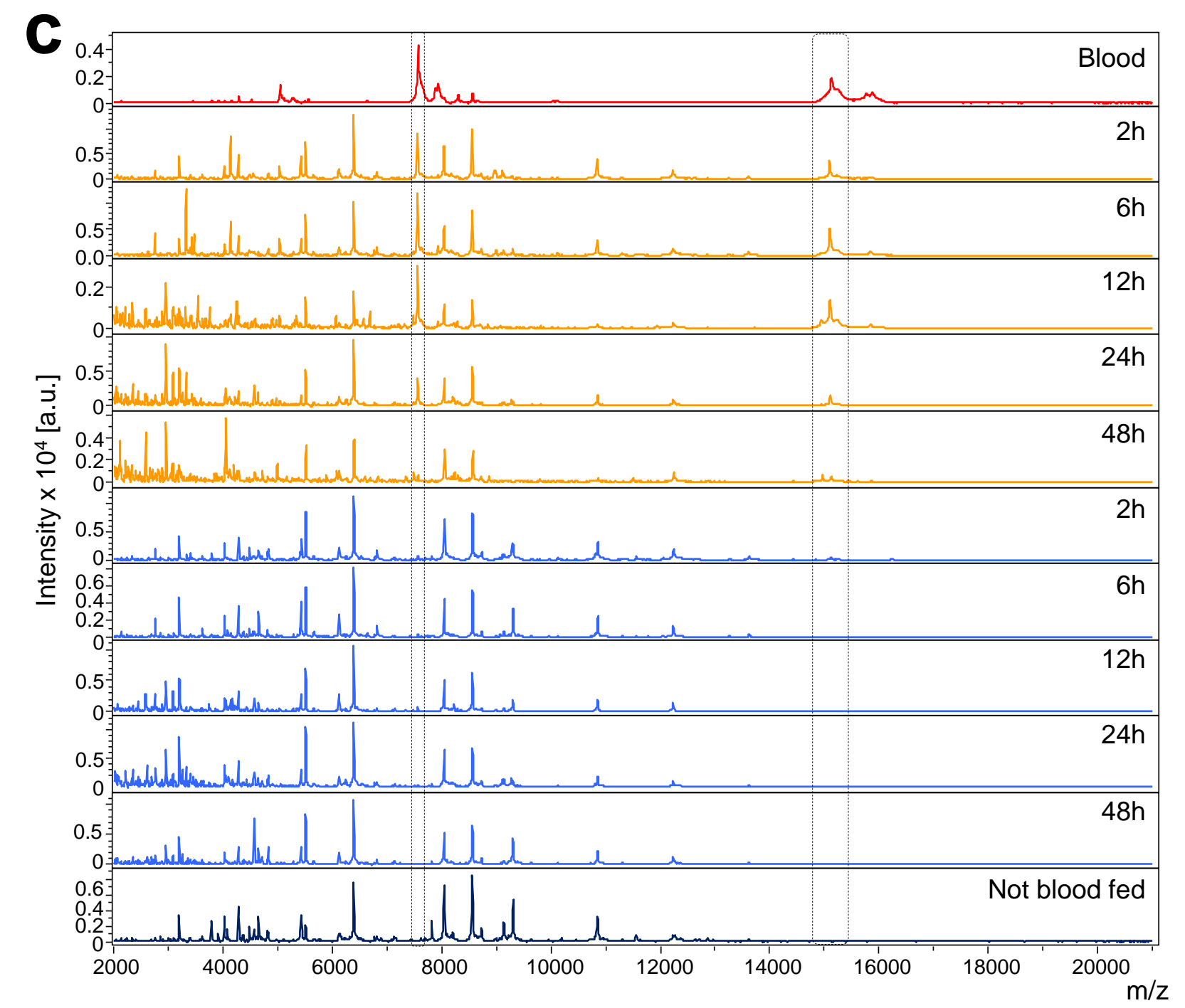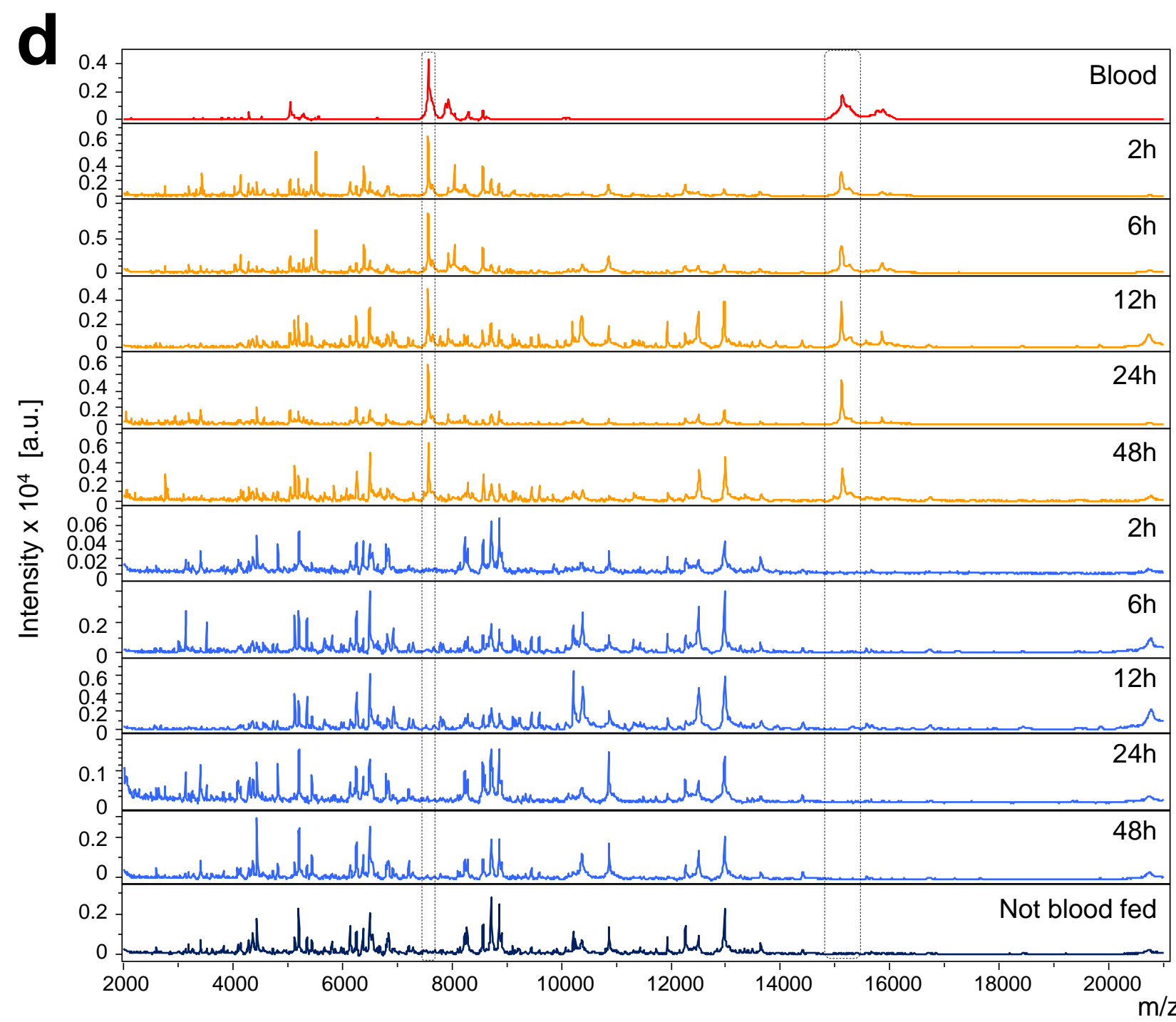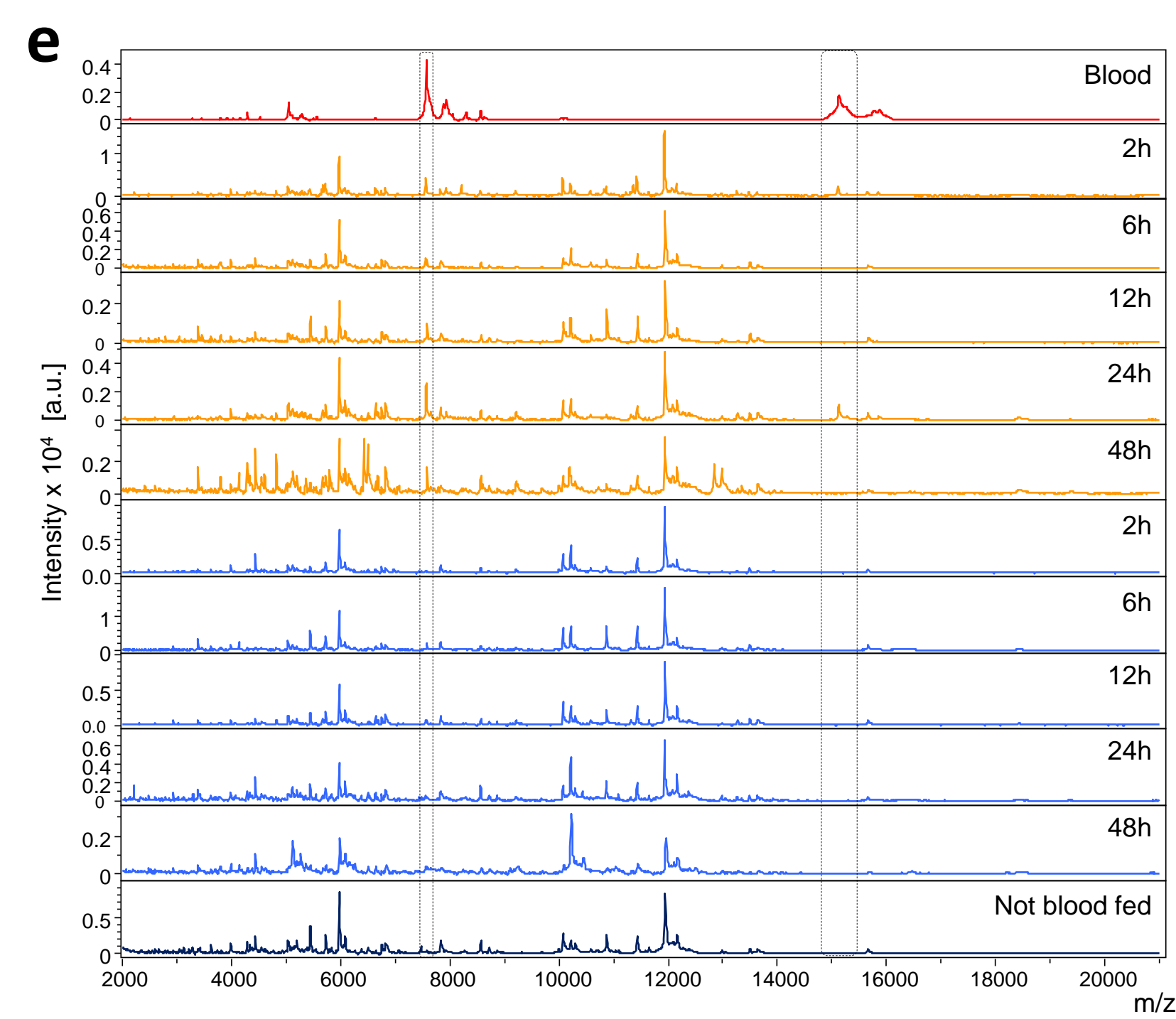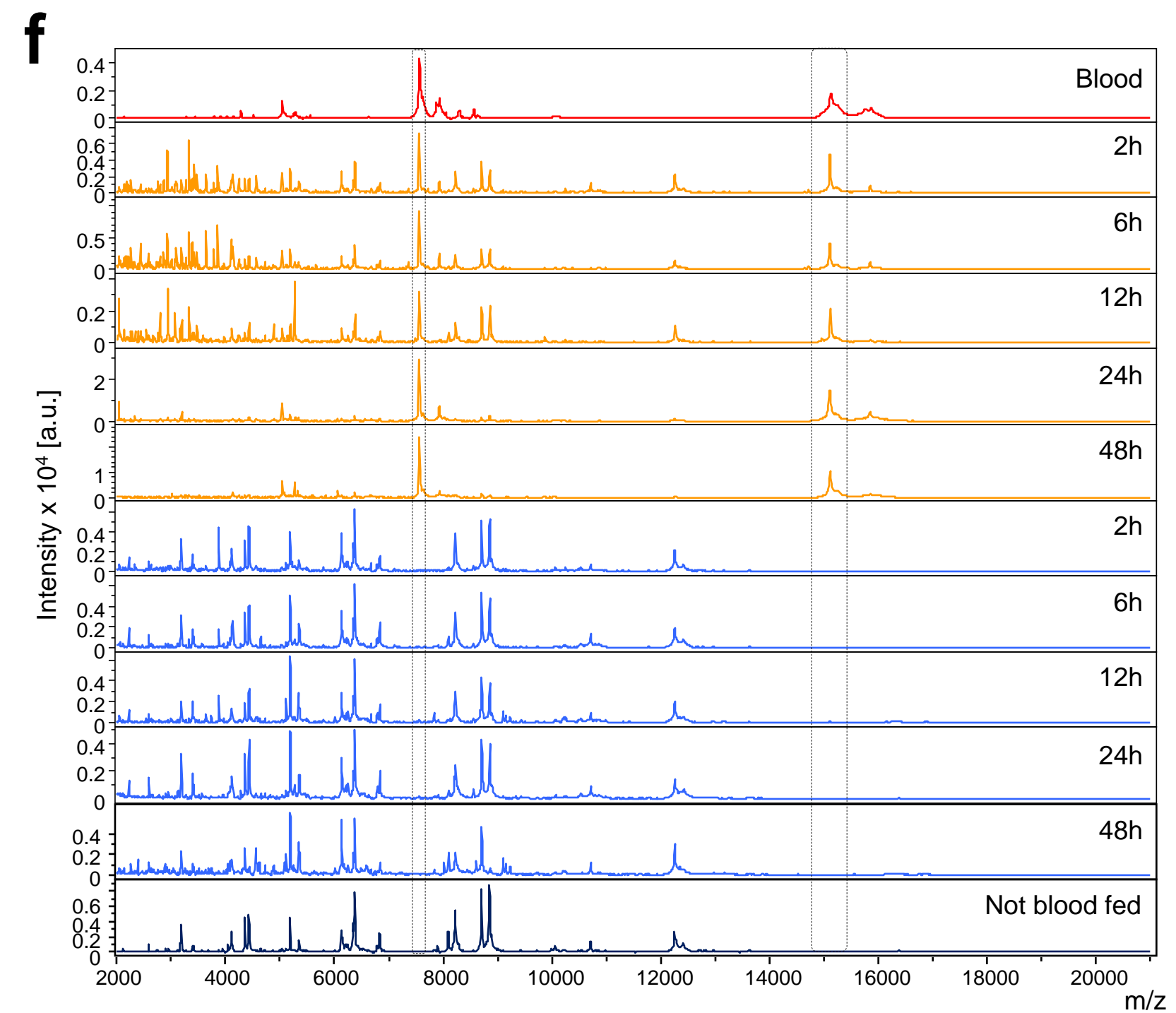

Supplement: Supplementary file 5 — Additional file 5: Figure S3. Comparison of MS spectra from heads (a, d), legs (b, e) and thoraxes (c, f) of (a, b, c0) Aedes aegypti (Bora) and (d, e, f) Anopheles coluzzii (Dkr), after blood engorgement and kinetically collected. MS spectra with (orange) or without (light blue) MS peaks shared with human blood MS profiles are presented. Dotted square highlight these shared MS peaks. MS spectra from human blood (red) were used as reference on each panel. MS spectra from respective species and body part of unfed mosquitoes were presented on each panel (dark blue). Time point collection post blood feeding were indicated on each MS spectra. a.u., arbitrary units; m/z, mass-to-charge ratio; h, hours. * Blood peak was not found in samples from 72h. [file 13071_2022_5361_MOESM5_ESM.pdf]

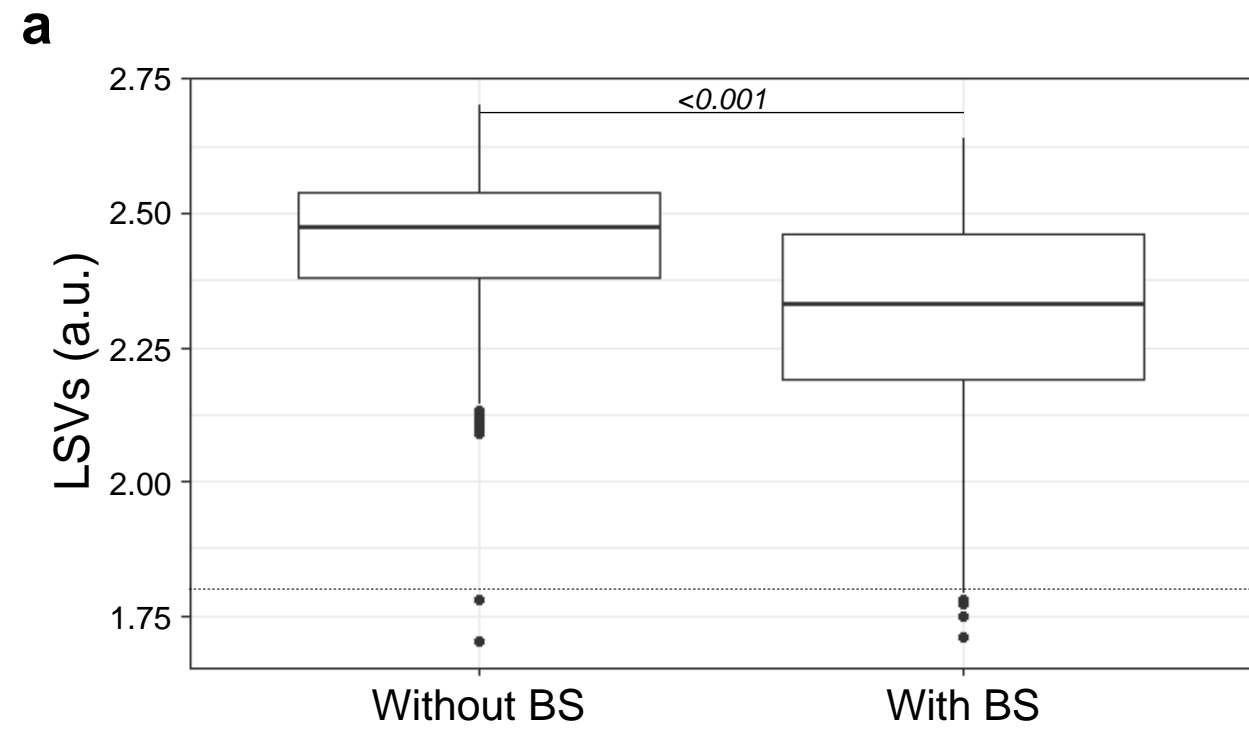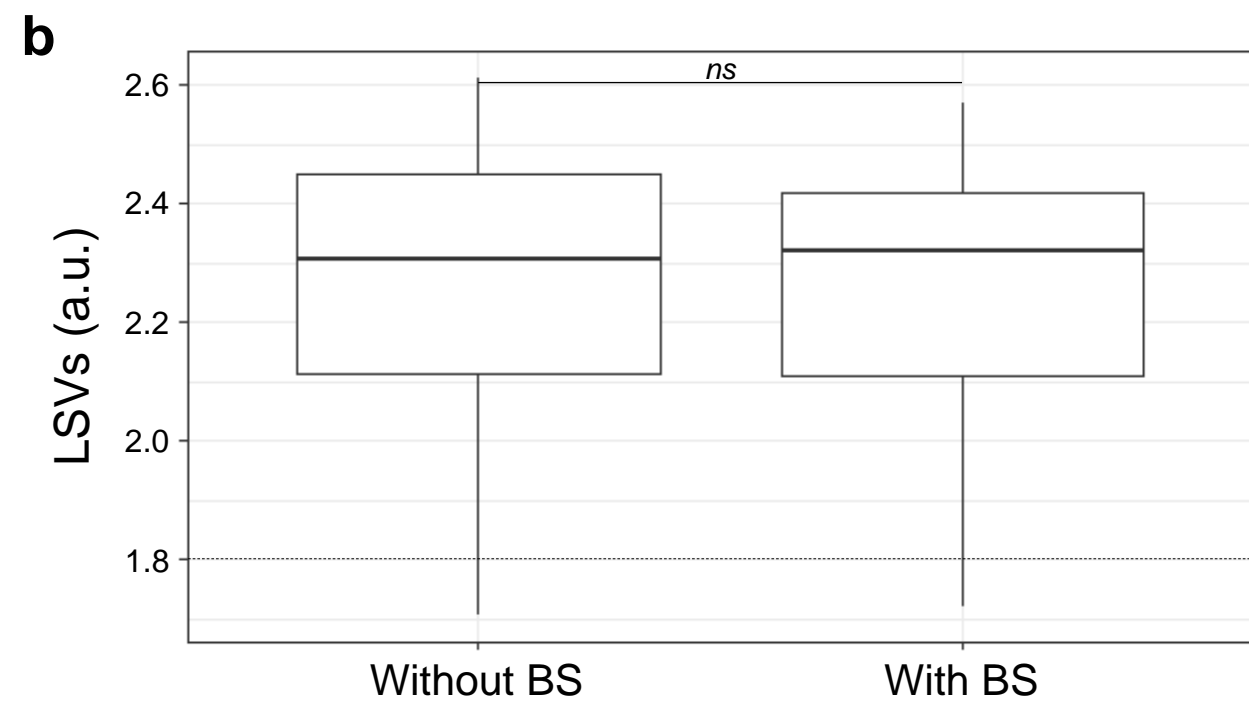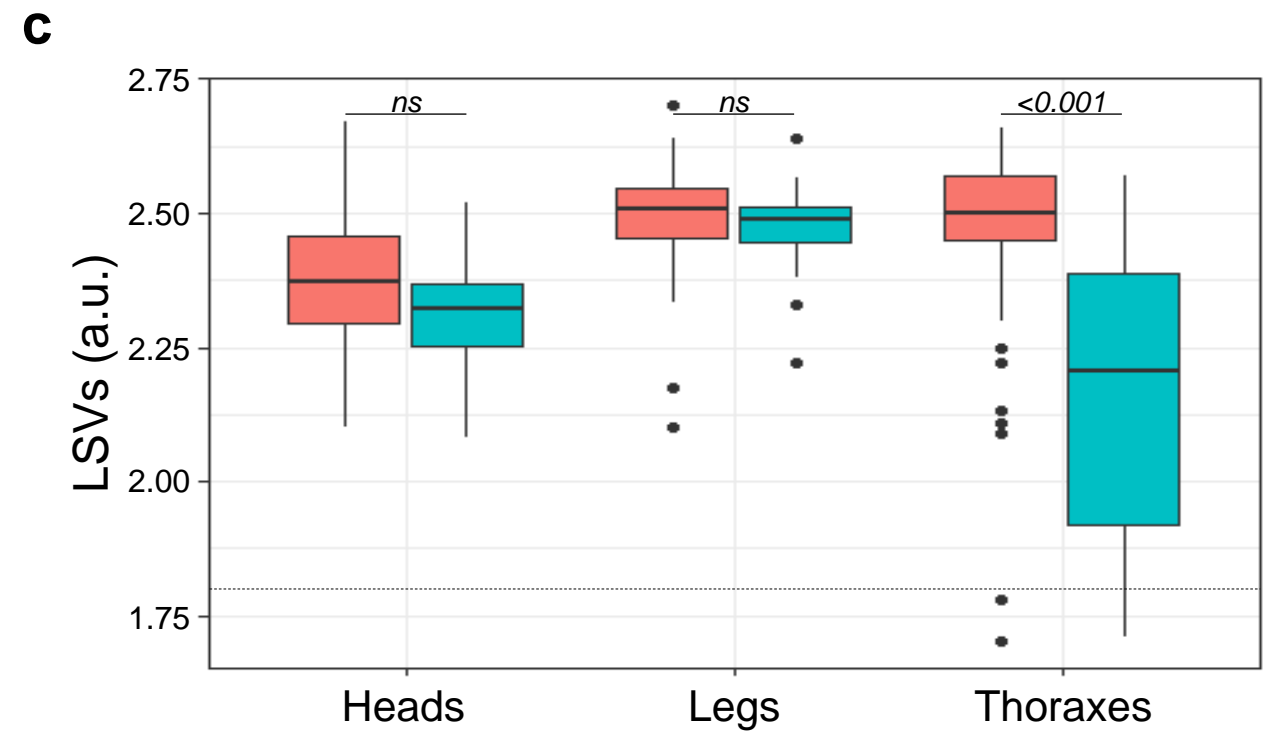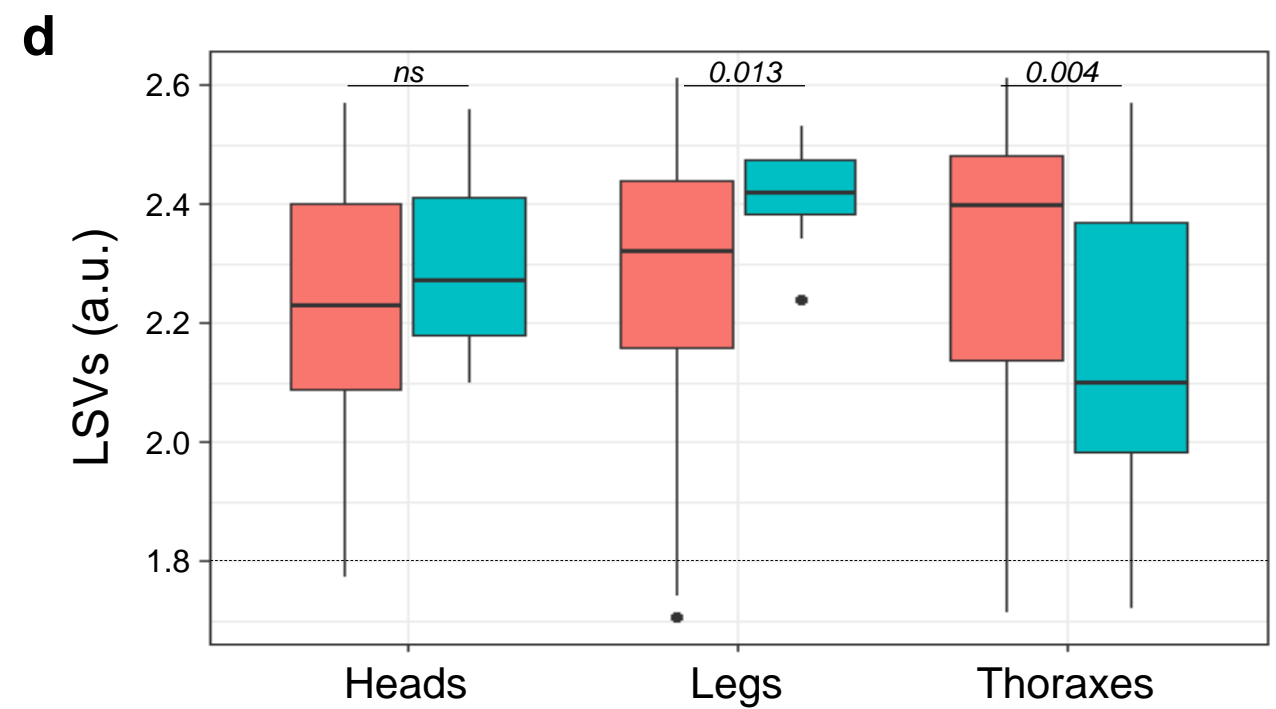

Supplement: Supplementary file 6 — Additional file 6: Figure S4. Comparison of LSVs from mosquito species with and without blood signature on their MS spectra. The LSVs obtained for (a, c) Ae. aegypti (Bora) and (b, d) An. coluzzii (Dkr) irrespective (a, b) or respective (c, d) to body parts classified according to the detection of MS peaks from blood origin were compared. Significant differences of LSVs obtained between MS spectra with (blue) and without (red) blood signature were indicated (Mann-Whitney test). Dashed line represents the threshold values (LSV ≥ 1.8), for relevant identification. All samples were homogenized using TL mode. a.u., arbitrary units; BS, blood signature; LSV, log score value; TL, TissueLyser. [file 13071_2022_5361_MOESM6_ESM.pdf]

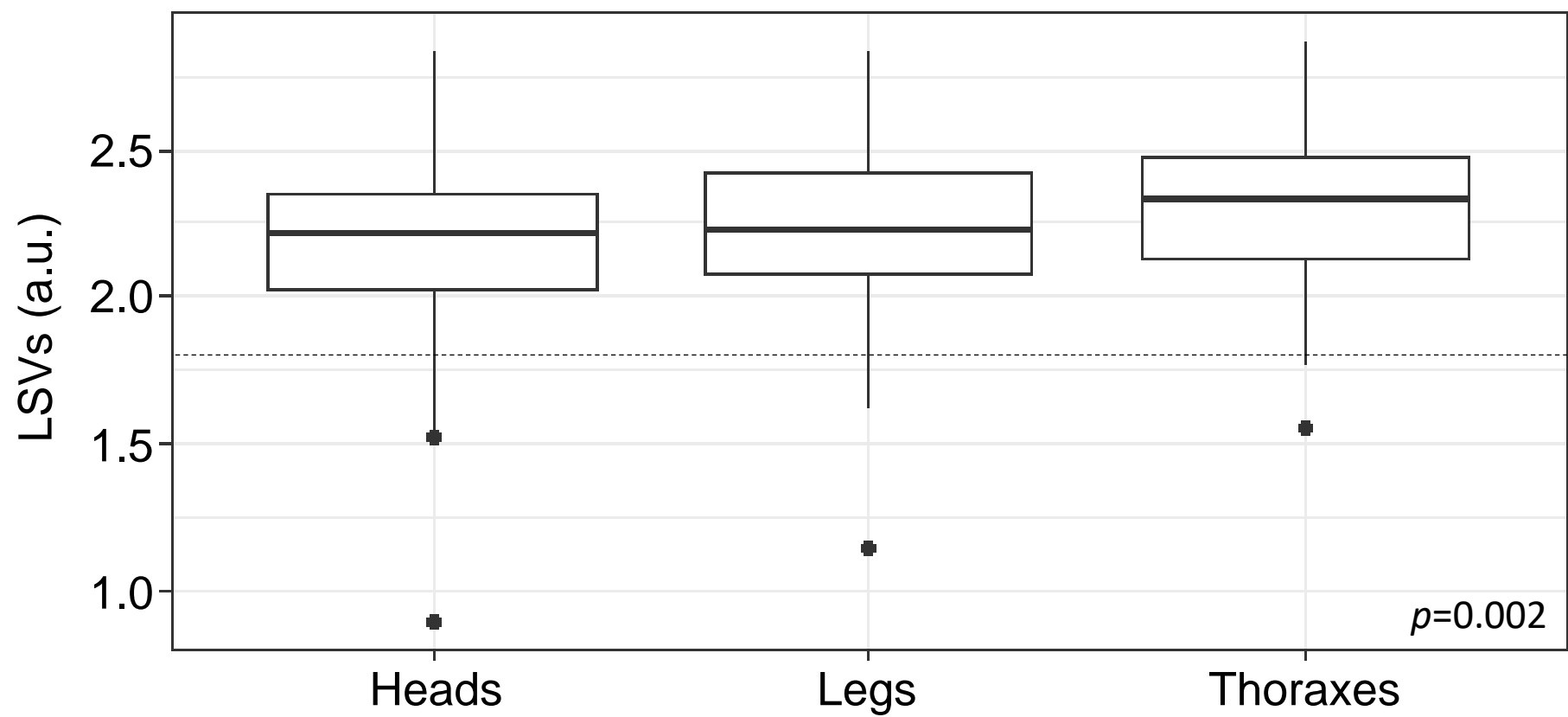

Supplement: Supplementary file 7 — Additional file 7: Figure S5. Comparison of LSVs between heads, legs and thoraxes, independently of the species, from laboratory or field origins. Significant differences of LSVs obtained between body parts were indicated in the right corner (Kruskal Wallis test). Dashed line represents the threshold values (LSV ≥ 1.8), for relevant identification. All samples were homogenized using TL mode. a.u., arbitrary units; LSV, log score value; TL, TissueLyser. [file 13071_2022_5361_MOESM7_ESM.pdf]
