# Supplementary material for: Enhanced procedures for mosquito identification by MALDI-TOF MS
Source: Parasit Vectors. 2022 Jun 30;15:240. doi: 10.1186/s13071-022-05361-0 (PMC9248115; doi:10.1186/s13071-022-05361-0)
Supplement: Supplementary file 8 — Additional file 8: Table S1. Time required for samples processing according to body part and homogenization mode. [file 13071_2022_5361_MOESM8_ESM.docx]

**Supplementary Table S1**. Time required for samples processing according to body part and homogenization mode.

|  | **Homogenization time (minutes)^b^** | | | | | | | | | | | |
| --- | --- | --- | --- | --- | --- | --- | --- | --- | --- | --- | --- | --- |
|  | **MP** |  |  | **PP** |  |  | **TL** |  |  | **Mean duration^c^** |  |  |
| **Number of samples^a^** | **Heads** | **Thoraxes** | **Legs** | **Heads** | **Thoraxes** | **Legs** | **Heads** | **Thoraxes** | **Legs** | **MP** | **PP** | **TL** |
| **1** | 1.0 ± 0.1 | 1.6 ± 0.4 | 1.1 ± 0.1 | 0.5 ± 0.1 | 1.0 ± 0.4 | 0.8 ± 0.3 | 3.0 | 3.0 | 3.0 | 1.2 ± 0.2 | 0.8 ± 0.3 | 3.0 |
| **5** | 4.9 ± 0.3 | 7.9 ± 0.7 | 5.3 ± 0.5 | 2.6 ± 0.3 | 4.8 ± 0.7 | 3.9 ± 0.5 | 3.0 | 3.0 | 3.0 | 6.0 ± 0.5 | 3.8 ± 0.5 | 3.0 |
| **24** | 23.5 ± 1.5 | 37.7 ± 3.5 | 25.4 ± 2.6 | 12.4 ± 1.5 | 23.2 ± 3.5 | 18.5 ± 2.6 | 3.0 | 3.0 | 3.0 | 28.9 ± 2.5 | 18.0 ± 2.5 | 3.0 |
| **48** | 46.9 ± 2.9 | 75.5 ± 7.0 | 50.7 ± 5.2 | 24.8 ± 2.9 | 46.4 ± 7.0 | 37.0 ± 5.2 | 3.0 | 3.0 | 3.0 | 57.7 ± 5.0 | 36.1 ± 5.0 | 3.0 |
| **72** | 70.4 ± 4.4 | 113.2 ± 10.4 | 76.1 ± 7.8 | 37.3 ± 4.4 | 69.6 ± 10.4 | 55.5 ± 7.8 | 6.0 | 6.0 | 6.0 | 86.6 ± 7.5 | 54.1 ± 7.5 | 6.0 |
| **96** | 93.9 ± 5.9 | 150.9 ± 13.9 | 101.4 ± 10.4 | 49.7 ± 5.9 | 92.8 ± 13.9 | 74.0 ± 10.4 | 6.0 | 6.0 | 6.0 | 115.4 ± 10.0 | 72.2 ± 10.0 | 6.0 |

^a^Homogenization time were measured for 1 and 5 samples, and estimated for the remaining (24 to 96).

^b^Duration in minutes are expressed by mean ± standard deviation.

^c^Mean for the three body parts per homogenization mode.

MP, micropipettes; PP, pellet pestles; TL, tissue lyser.
