# Supplementary material for: Enhanced procedures for mosquito identification by MALDI-TOF MS
Source: Parasit Vectors. 2022 Jun 30;15:240. doi: 10.1186/s13071-022-05361-0 (PMC9248115; doi:10.1186/s13071-022-05361-0)
Supplement: Supplementary file 9 — Additional file 9: Table S2. Proportion and number of specimens with blood signature in the MS spectra [file 13071_2022_5361_MOESM9_ESM.docx]

**Table S2**. Proportion and number of specimens with blood signature in the MS spectra

|  |  | ***Ae. aegypti* (Bora)** | | | | | | ***An. coluzzii* (Dkr)** | | | | | |
| --- | --- | --- | --- | --- | --- | --- | --- | --- | --- | --- | --- | --- | --- |
| **Time (h)^*^** | **N^#^** | **Heads** | | **Legs** | | **Thorax** | | **Heads** | | **Legs** | | **Thorax** | |
|  |  | % | (n) | % | (n) | % | (n) | % | (n) | % | (n) | % | (n) |
| **2** | 20 | 30 | 6 | 20 | 4 | 30 | 6 | 15 | 3 | 10 | 2 | 40 | 8 |
| **6** | 20 | 20 | 4 | 25 | 5 | 50 | 10 | 20 | 4 | 15 | 3 | 35 | 7 |
| **12** | 20 | 20 | 4 | 25 | 5 | 50 | 10 | 20 | 4 | 5 | 1 | 15 | 3 |
| **24** | 20 | 15 | 3 | 0 | 0 | 35 | 7 | 35 | 7 | 20 | 4 | 35 | 7 |
| **48** | 20 | 5 | 1 | 5 | 1 | 5 | 1 | 5 | 1 | 25 | 5 | 10 | 2 |
| **72** | 10 | 0 | 0 | 0 | 0 | 0 | 0 | 0 | 0 | 0 | 0 | 0 | 0 |
| **Total** | 110 | 16.4 | 18 | 13.6 | 15 | 30.9 | 34 | 17.3 | 19 | 13.6 | 15 | 24.5 | 27 |

^*^Time of collection after blood feeding (engorgement) of mosquitoes using Hemotek.

^#^N: number of sample per body part and time; h: hour
